# Seasonal Variation of Butterfly Diversity in Subtropical Urban Forests of Nepal

**DOI:** 10.1002/ece3.73936

**Published:** 2026-07-03

**Authors:** Mahamad Sayab Miya, Apeksha Chhetri, Bandana Subedi, Pratiksha Sharma, Sanjaya Raj Tamang, Shristee Panthee, Hasina Miya

**Affiliations:** ^1^ Department of Biological Sciences Western Kentucky University Bowling Green Kentucky USA; ^2^ Department of Entomology and Plant Pathology University of Arkansas Fayetteville Arkansas USA; ^3^ Center for Agricultural Data Analytics University of Arkansas Fayetteville Arkansas USA; ^4^ Institute of Forestry, Pokhara Campus Tribhuvan University Pokhara Nepal; ^5^ Université du Québec en Abitibi‐Témiscamingue Rouyn‐Noranda Quebec Canada; ^6^ College of Natural Resource Management Agriculture and Forestry University Katari Nepal; ^7^ Kathmandu Forestry College Tribhuvan University Kathmandu Nepal; ^8^ Leibniz Institute for Vegetable and Ornamental Crops (IGZ) e.V. Großbeeren Germany; ^9^ Institute of Biodiversity Friedrich‐Schiller Universität Jena Jena Germany

**Keywords:** bioindicator, Lepidoptera, Nymphalidae, Pokhara, seasonality, subtropical forest

## Abstract

Butterflies exhibit seasonal dynamics in diversity and population in response to bioclimatic factors. However, their seasonal diversity remains poorly understood in urban landscapes. Therefore, we examined how butterfly diversity varies across seasons in the urban forests of Pokhara City, located in Nepal's subtropical region. We selected three urban forests—Banpale, Bhadrakali, and Shanti Ban Batika—to sample butterflies. We employed the Pollard Walk Survey method to collect data across all four seasons and analyzed various diversity indices, community composition, and indicator species. We also applied generalized linear mixed models (GLMMs) to assess the effects of precipitation and temperature on species richness and abundance. In total, 197 butterfly species, comprising 3344 individuals from six families, were recorded. The diversity and community composition differed significantly across seasons, with richness, Shannon, and Simpson diversities peaking during the monsoon and abundance peaking in the pre‐monsoon. Winter recorded the lowest species richness, diversity, and abundance. Species like *Pieris canidia*, *Eurema hecabe*, *Ypthima baldus*, and *Junonia iphita* contributed most to the differences across seasons. *Anthene emolus* was associated with the pre‐monsoon season, *Lethe kansa* and *Athyma ranga* were confined to winter, and several other species served as seasonal indicators. The family Nymphalidae dominated the butterfly community, with different families exhibiting notable seasonal variation in richness and abundance. Additionally, GLMMs found that precipitation alone has a significant positive effect on species richness, while the combined effect of precipitation and temperature negatively impacts both richness and abundance. This study highlights the importance of seasonality in shaping butterfly diversity and community composition in urban forests, offering essential insights for their conservation in urban landscapes.

## Graphical abstract

Butterflies show seasonal fluctuations in diversity and population in response to bioclimatic factors. Studying butterflies in three urban forests in the subtropical region of Nepal, we found that the diversity and community composition differed significantly across seasons, with richness and diversity peaking during the monsoon and abundance peaking in the pre‐monsoon. Additionally, GLMMs revealed that precipitation alone has a significant positive effect on species richness, while the combined effect of precipitation and temperature negatively impacts both richness and abundance in the study area.

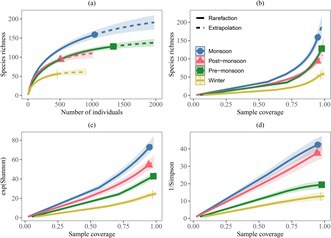

## 1 Introduction

Seasonality plays a vital role in shaping the population dynamics and diversity of insects (da Silva et al. 2011), which make up more than half of the world's terrestrial species (Stork 2018). Insect populations usually peak during the rainy season and decline in dry periods (Wolda 1988). Therefore, understanding these seasonal fluctuations is essential for their conservation amid escalating climate variability (John et al. 2024). Butterflies are among the most extensively studied insect groups worldwide, with more than 19,500 species described (Kawahara et al. 2023; van Nieukerken et al. 2011). They play a crucial role in plant pollination and in the food web, serving as an excellent food source for spiders, reptiles, amphibians, birds, and carnivorous insects (Tiple et al. 2006; Tiple and Bhagwat 2023). Furthermore, because of their high sensitivity to habitat alterations and climate change, they are considered bioindicators of terrestrial ecosystems (Hill et al. 2021; Stuhldreher and Fartmann 2018; Syaripuddin et al. 2015). Butterflies have been widely used to detect impacts of climate change (Remadevi et al. 2018), forest restoration or fragmentation (Legal et al. 2020; Pignataro et al. 2023), and shifts in biodiversity (Whitworth et al. 2018).

Butterflies are well‐suited for studying how seasonality impacts insect diversity, as some species are found year‐round while many are confined to specific seasons (Kunte 1997). The seasonal behaviors of butterflies are influenced by various factors, including rainfall, temperature, humidity, predator abundance, food availability, and habitat (Bhusal and Khanal 2009; Freire et al. 2014; Herrando et al. 2019). Generally, butterflies remain in the pupal stage during the dry season and emerge when rains begin (Janzen 1987). Several species enter diapause as larvae inside pupal cocoons during the dry season and pupate during the next rainy season (Aiello 1992). In contrast, some butterflies breed continuously and expand or contract their geographic ranges (Jones and Rienks 1987). The gradual increase in temperature influences insect behavior, physiology, distribution, and species interactions. Simultaneously, the rise in extreme events like heatwaves, cold spells, wildfires, droughts, and floods further affects these aspects (Harvey et al. 2023). Recent climate fluctuations have significantly affected butterfly habitats and population dynamics (Pinkert et al. 2025), as evidenced by a sharp worldwide decline in butterfly richness and abundance (Hill et al. 2021; Wagner et al. 2021). Therefore, understanding the seasonal patterns of butterflies and the influence of environmental factors on their diversity is essential for conservation efforts.

Nepal has six climatic zones, ranging from tropical in the southern plains to tundra or nival, snow‐covered zones in the north (Paudel et al. 2021). This variation in geography and climate across the country supports about 695 described butterfly species (Kc et al. 2025; van der Poel and Smetacek 2022). However, the country's biodiversity research priorities are skewed toward megafauna, often neglecting invertebrates or insects like butterflies. As a result, documentation of butterfly diversity across the country remains limited to specific regions, habitats, or seasons. Some studies on seasonal or habitat‐specific butterfly diversity are concentrated in tropical areas (e.g., Khanal 2009; Miya et al. 2025; Mulmi et al. 2025; Oli et al. 2023; Tamang et al. 2019), and others in the subtropical (1000–2000 m) to temperate regions (e.g., Bhusal and Khanal 2009; Kc and Sapkota 2024; Khanal 2018, 2020; Khanal et al. 2012; Neupane and Miya 2021). There is a notable knowledge gap regarding butterfly seasonality in the urban forests across the country. Few studies have explored butterfly diversity in urban forest habitats, for instance, in the Kathmandu Valley (Aryal et al. 2026; Bhatta 2023; Khanal et al. 2013; Oli and Sharma 2019; Shrestha et al. 2018) and in the Pokhara Valley (Kc 2025). Urban forests are vital habitats for butterflies (Huang et al. 2024; Koh and Sodhi 2004). They face high levels of disturbance in these landscapes and exhibit seasonal and site‐based variation (Fang et al. 2023; Lin et al. 2024; Ombugadu et al. 2024), underscoring the importance of understanding their seasonal dynamics in urban environments.

The present study aimed to address the knowledge gap about butterfly seasonality in urban landscapes by examining butterflies throughout all seasons in urban forests in Pokhara City, Pokhara Valley, a subtropical region of Nepal. The three main urban forests in the city—Banpale Forest, Bhadrakali Forest, and Shanti Ban Batika Forest—were chosen for the study. To our knowledge, no published literature lists butterflies from these forests. A recent study by Kc (2025) documented butterflies in the northwest part of the city (Methlang Forest), which connects to other forest patches to the north and west. This study, based on an opportunistic survey, reported species richness (not abundance) and variations in richness across months. In contrast, our study offers a comprehensive view of butterfly seasonality by reporting both richness and abundance in more isolated urban forests within the city. These three forests experience high human interference and contain a mix of forest and anthropogenic habitats. For example, Banpale Forest includes forest area, nurseries, buildings, open grounds, and gardens, which can buffer butterfly dynamics across seasons (Lourenço et al. 2020) and may reveal different findings. Therefore, this study has broad applications for understanding butterfly seasonality in subtropical urban landscapes.

Given prior knowledge of the role of seasonality in insect diversity and population dynamics (Habel et al. 2018; Kunte 1997; da Silva et al. 2011) and of butterfly diversity differences across urban landscapes (Aryal et al. 2026; Fang et al. 2023; Lin et al. 2024), we also assumed that butterflies show seasonal variation in subtropical urban forests. We hypothesized that wetter seasons are associated with greater butterfly species richness, abundance, and diversity, and that these patterns vary across taxonomic (family) levels. Therefore, this study aims to investigate seasonal variation in (a) overall butterfly diversity, (b) community composition, and (c) family‐wise species richness and abundance. We also examined the effects of two climatic factors (monthly precipitation and temperature) on butterfly species richness and abundance. The findings of our study could serve as a valuable information for butterfly conservation in the study area and more widely in subtropical regions.

## 2 Materials and Methods

### 2.1 Study Area

**FIGURE 1:**
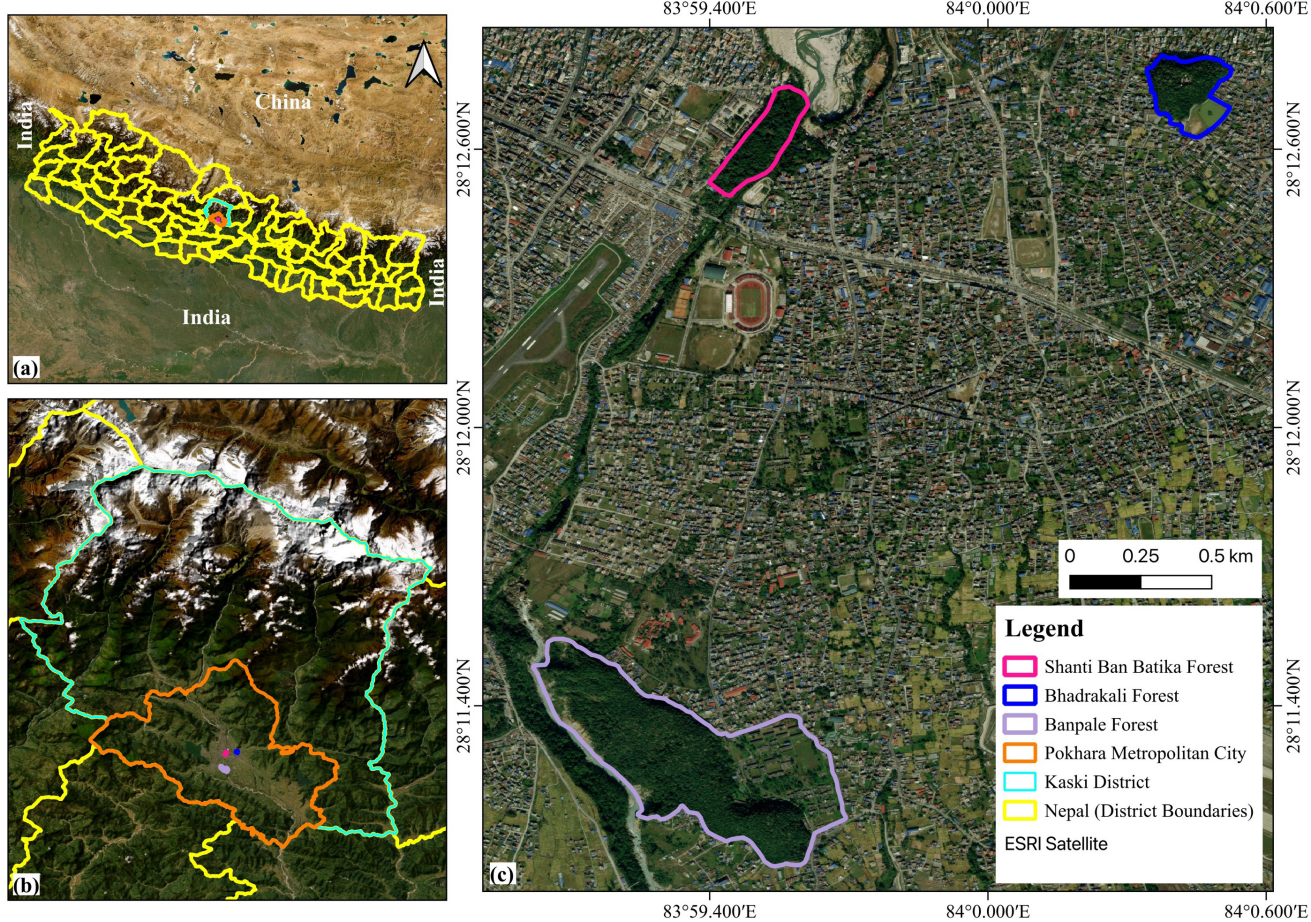
Map of the study area showing the boundaries of (a) Nepal (district level), (b) Kaski District (cyan color) and Pokhara Metropolitan City (orange), and (c) three urban forests: Banpale (cold purple), Bhadrakali (blue), and Shanti Ban Batika (pink). The map was created in QGIS (version 3.36.2‐Maidenhead).

The study was carried out in three urban forests within Pokhara City, Kaski District, a subtropical region of Nepal. These forests include Banpale Forest (BF), Bhadrakali Forest (BKF), and Shanti Ban Batika Forest (SBBF) (Figure 1). Among them, BF (28°11′17.89″ N, 83°59′21.30″ E) is the largest, covering 46.85 ha (Baral et al. 2022). It is situated on the premises of the Institute of Forestry, Pokhara Campus (IOF‐PC), which features buildings, gardens, nurseries, open playgrounds, shrubs with scattered trees in the southeastern part, and primary forest in the rest of the area. It supports a rich biodiversity, with 331 documented plant species (Miya and Gautam 2021), 125 bird species (Baral et al. 2022), and 20 mammal species, including 10 bat species (Bhattarai et al. 2021; Bist et al. 2017). Bhadrakali Forest (28°12′42.99″ N, 84° 0′25.83″ E) spans ~6.58 ha in the southeastern part of the city. Both BF and BKF forests are primarily dominated by *Schima‐* *Castanopsis* species. Shanti Ban Batika Forest (28°12′36.82″ N, 83°59′30.70″ E) covers ~5.34 ha and is situated near the center of the city. The forest features dense mixed trees of *Diploknema butyracea* , *Osmanthus fragrans* , and *Diospyros melanoxylon*. All three forests host invasive plant species such as *Bidens pilosa* , *Lantana camara* , *Ageratina adenophora* , and *Ageratum houstonianum* , which attract butterflies for nectar. BF and SBBF, owned and managed by IOF‐PC, serve as important sites for research and learning for students and researchers. The study area experiences four main seasons based on rainfall: pre‐monsoon (March–May), monsoon (June–September), post‐monsoon (October–November), and winter (December–February). Generally, Pokhara receives about 4000 mm of annual precipitation, which varies seasonally: pre‐monsoon (< 500 mm), monsoon (< 3000 mm), post‐monsoon (< 150 mm), and winter (~100 mm). The average annual temperature is 21°C, with seasonal variations: 22°C during pre‐monsoon, 26°C in monsoon, 23°C in post‐monsoon, and 14°C in winter (Gautam et al. 2019).

### 2.2 Data Collection

**FIGURE 2:**
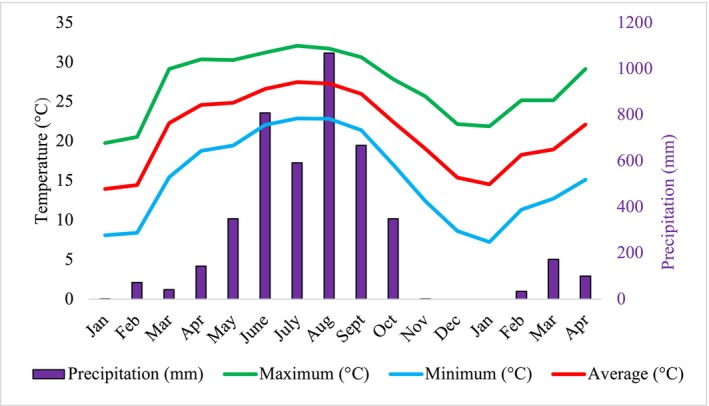
Bar and line plots illustrating monthly precipitation and temperature in Pokhara City for 2022 (Jan–Dec) and 2023 (Jan–Apr). Months are on the X‐axis, temperature (°C) on the left Y‐axis, and precipitation (mm) on the right Y‐axis. The bars represent monthly precipitation variations. The green, red, and sky‐blue lines indicate the maximum, average, and minimum temperatures, respectively.

We followed the Pollard Walk Survey method (Pollard 1977) with slight modifications to the transect length for butterfly data collection. Among the several techniques employed for butterfly sampling (Royer et al. 1998; Henry et al. 2015; Kral et al. 2018; Freitas et al. 2020; Bruschini et al. 2024), the Pollard Walk is widely used for long‐term monitoring. A total of 19 fixed transects, each 200 m long, were established with gaps of about 50–100 m between them. Of these, 11 transects were in BF, and four each in BKF and SBBF, aimed to cover core forest areas, edges, buildings, remaining open grounds, and bushes. The study began in 2017 with opportunistic surveys and was developed into a systematic study from March 2022 to February 2023. We sampled butterflies for a year, covering all four seasons: pre‐monsoon, monsoon, post‐monsoon, and winter. Each transect was visited once a month, resulting in 12 replications per transect. Butterflies were observed within an imaginary 5 m × 5 m × 5 m box, walking at a steady pace along a transect, meaning species were observed across 2.5 m on each side of the transect, 5 m above, and 5 m ahead. Weather conditions were taken into account, with data collection avoided on rainy days, and sampling conducted from 10:00 to 15:00 to maximize butterfly detection. During each field visit, we recorded the species names and their abundance (count). Species were identified in the field using butterfly guidebooks (Smith 2011; Smith et al. 2016), and those that were difficult to identify were recorded as unknown, noting their morphological features, such as dominant color, wing shape, wing pattern, approximate body size, and ocelli. For better identification, we photographed butterflies using smartphones and cameras. Some butterflies were challenging to identify and photograph due to their erratic flight. In this case, we used butterfly nets to capture them, photographed, and then released immediately. No harm was done to the butterflies during this process. The unidentified butterflies were later confirmed by comparing them with images and descriptions in guidebooks (Smith 2011; Smith et al. 2016), online sources, published literature (e.g., Neupane and Miya 2021; Tamang et al. 2019; van der Poel and Smetacek 2022), and expert consultations. The IUCN status of butterflies was accessed through the IUCN Red List of Threatened Species (IUCN 2025). As we focused on seasonal variation, we obtained monthly precipitation (rainfall) and temperature data (ranging from January 2022 to Apr 2023), recorded at the nearby weather station: Pokhara Airport. This data was acquired from the Department of Hydrology and Meteorology in Babarmahal, Kathmandu, Nepal, and is shown in Figure 2.

### 2.3 Data Analysis

We used R version 2026.01.0 (R Core Team 2026) to analyze butterfly data—computed Hill diversity, Pielou's Evenness Index (J), nonmetric multidimensional scaling (NMDS), similarity percentage (SIMPER), indicator species (IndVal), and generalized linear mixed models (GLMMs). First, we determined the Hill diversity (Hill number;*q*) using the “iNEXT” package, with 200 bootstrap replications and 95% confidence intervals (CIs) (Chao et al. 2020; Hill 1973; Hsieh et al. 2016) to compare seasonal butterfly diversity. This method uses rarefaction and extrapolation to assess diversity based on the reference sample. Using individual‐based abundance data (Chao et al. 2014), we measured three types of Hill diversity: species richness (*q* = 0), Hill Shannon diversity (*q* = 1, also called exponential of Shannon entropy or Shannon Index), and Hill Simpson diversity (*q* = 2, also called inverse of Simpson concentration or Simpson Index). The Hill number (effective number of species) is more statistically robust than other diversity indices and combines species richness with relative abundance (Chao et al. 2014; Roswell et al. 2021). Species richness only considers presence or absence, ignoring abundance and treating all species equally. Hill Shannon diversity (hereafter exponential Shannon Index) is based on proportional counts of species abundance, reflecting the effective number of common species in the community. Hill Simpson diversity (hereafter inverse Simpson Index) is estimated from counts of dominant species and indicates the effective number of dominant species. Higher ‘*q*’ values indicate greater richness and diversity. When confidence intervals overlap, the difference between groups is not statistically significant. Hill numbers are widely used in ecological research (Fang et al. 2023; Ricotta and Feoli 2024). To evaluate how individuals are distributed within the community, we calculated Pielou's Evenness Index (*J*) using the “vegan” package (Dixon 2003).

Next, to compare butterfly community composition across seasons, NMDS based on Bray–Curtis distance and analysis of similarities (ANOSIM) was performed using the “vegan” package (Dixon 2003). The contribution of each species to seasonal differences was assessed with similarity percentage analysis (SIMPER) based on Bray–Curtis dissimilarities, using the “simper()” function. Indicator species analysis (IndVal) was also conducted to identify species strongly associated with specific seasons, utilizing the “indicspecies” package with 999 permutations (de Cáceres and Legendre 2009). The indicator value ranges from 0 to 1, with 1 indicating a strong association with a particular season.

Furthermore, to assess the effect of monthly precipitation and average temperature on species richness and abundance, GLMMs were employed (Bolker et al. 2009). Species richness and abundance served as response variables, while precipitation and temperature were predictors, with sites and months treated as random factors. The predictors were standardized to have a mean of 0 and a standard deviation of 1. For the species richness model, Poisson regression was applied, and negative binomial regression was used for the abundance model to address overdispersion. The “lme4” and “glmmTMB” packages were used for this analysis (Bates et al. 2026; Brooks et al. 2026). The Akaike Information Criterion (AIC) was used to compare models and select the best one (Akaike 1974), and model fit was evaluated using the “DHARMa” package (Hartig 2024). Results were visualized using packages such as “ggplot2,” “ggthemes,” “patchwork,” “UpSetR,” and “sjPlot” (Arnold 2025; Conway et al. 2017; Lüdecke 2025; Pedersen 2025; Wickham 2016).

## 3 Results

We documented 197 butterfly species comprising 3344 individuals from six families across three urban forests. Among them, we recorded 1972 individuals belonging to 194 species in Banpale, 697 individuals of 74 species in Bhadrakali, and 675 individuals of 65 species in Shanti Ban Batika (Appendix A). Banpale hosts the highest number of unique species at 105, while 47 species were shared among all three forests (Figure S1 and Table S1). Butterfly images are shown in Appendix B (Images 1–167). For the forest‐specific butterfly diversity (refer to Figure S2 and Table S1).

### 3.1 Seasonal Variation of Butterfly Diversity

**FIGURE 3:**
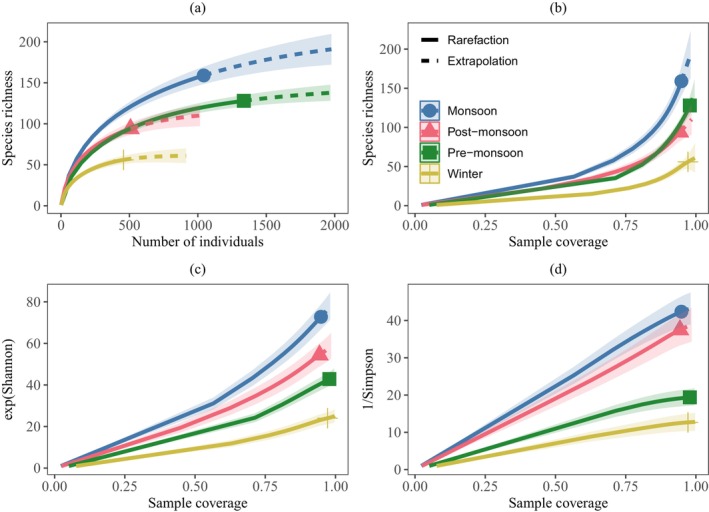
Rarefaction and extrapolation curves for butterfly species richness and diversity across different seasons. (a) Species accumulation curve based on the number of individuals; (b) species richness, (c) exponential Shannon Index, and (d) inverse Simpson Index, all three based on sample coverage. The solid curves represent rarefaction, the dashed lines indicate extrapolation, and the shaded areas show the corresponding 95% confidence intervals. There is no statistically significant difference in the diversity indices between seasons when the confidence intervals overlap at *p* < 0.05.

The observed species richness significantly varied between seasons, except for pre‐monsoon and post‐monsoon. It was significantly highest during the monsoon (159.00 ± 17.37) and lowest in winter (56.00 ± 7.18). The exponential Shannon Index also showed significant differences across all seasons, with the highest in monsoon (72.78 ± 3.24) and the lowest in winter (24.01 ± 1.63). Similarly, the inverse Simpson Index varied across seasons, except during the monsoon and post‐monsoon seasons. It was highest in the monsoon (42.36 ± 2.42) and lowest in winter (12.66 ± 1.26) (Table 1, Figure 3). The abundance was maximum in pre‐monsoon (*N* = 1336), followed by monsoon (*N* = 1043), and lowest in winter (*N* = 457). Evenness was highest in post‐monsoon (0.87), followed by monsoon (0.84), and lowest in pre‐monsoon (0.77) (Table 1). For the forest‐wise seasonal variation of butterfly diversity (see Figures S3–S5 and Tables S2–S4).

**TABLE 1:** Hill diversity (*q*, Mean ± SE), abundance (*N*), sample coverage (SC), and evenness (*J*) of butterflies across the seasons.

Seasons	*q* = 0	*q* = 1	*q* = 2	*N*	SC	*J*
Pre‐monsoon	128.00 ± 10.36	42.85 ± 1.72	19.39 ± 1.11	1336	0.97	0.77
Monsoon	159.00 ± 17.37	72.78 ± 3.24	42.36 ± 2.42	1043	0.94	0.84
Post‐monsoon	94.00 ± 15.96	54.41 ± 3.09	37.49 ± 2.50	508	0.94	0.87
Winter	56.00 ± 7.18	24.01 ± 1.63	12.66 ± 1.26	457	0.97	0.78

### 3.2 Butterfly Community Composition Across Seasons

**FIGURE 4:**
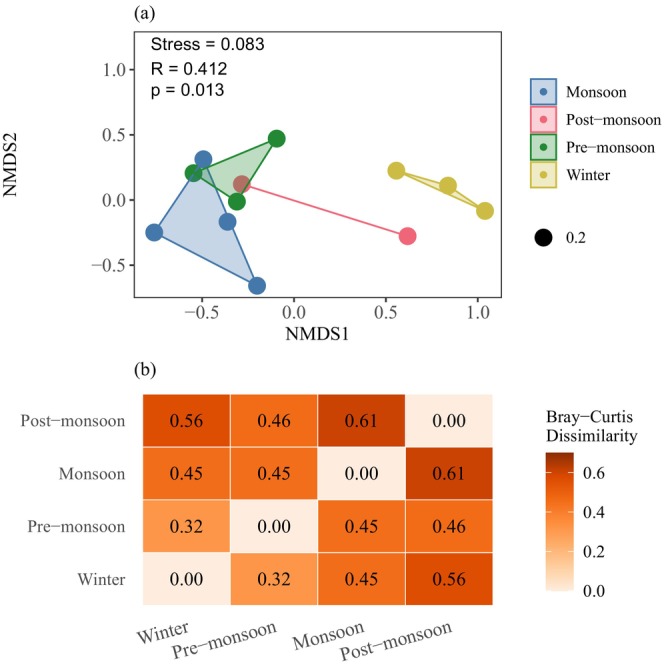
(a) Nonmetric multidimensional scaling (NMDS) ordination of all sampling units (abundance) and (b) Bray–Curtis dissimilarity heatmap, indicating the relative differences in butterfly community composition between seasons (*p <* 0.05).

**FIGURE 5:**
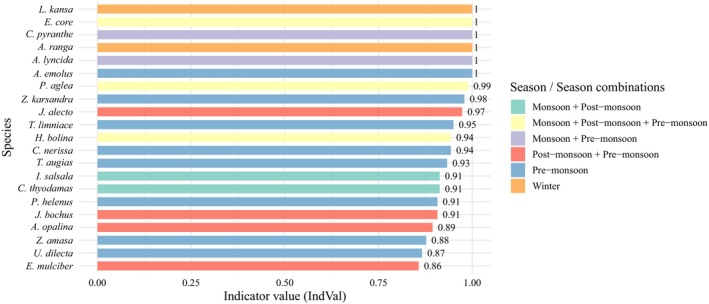
Bar plot showing the significant indicator species associated with a season or combinations of seasons (*p <* 0.05).

The NMDS ordination revealed a significant difference in butterfly community composition between winter and the other three seasons, post‐monsoon and monsoon/winter, with partial similarity between pre‐monsoon and monsoon/post‐monsoon (stress = 0.083 and *p* = 0.013) (Figure 4a). The greatest dissimilarity was observed between monsoon and post‐monsoon (Bray‐Curtis = 0.61), followed by post‐monsoon and winter (Bray‐Curtis = 0.56) (Figure 4b). For the NMDS ordination analysis of each site (see Figures S6–S8). The SIMPER analysis identified species such as *Pieris canidia*, *Eurema hecabe*, *Ypthima baldus*, and *Junonia iphita* as the top contributors to seasonal dissimilarity (Table S5). A total of 21 species were found to be significantly associated with individual seasons or combinations of seasons. For example, *Anthene emolus* was strongly associated with pre‐monsoon (IndVal = 1), while *Lethe kansa* and *Athyma ranga* served as strong indicators of winter (IndVal = 1). Other remaining species were indicators of one or more seasons (Figure 5 and Table S6).

### 3.3 Seasonal Variation of Family‐Wise Butterfly Species Richness and Abundance

We found that Nymphalidae had the highest species richness with 75 species, followed by Lycaenidae with 47 species, and Riodinidae with the fewest (5 species). Similarly, abundance was greatest in Nymphalidae (*N* = 1647), followed by Pieridae (*N* = 772), and lowest in Riodinidae (*N* = 43) (Table S7). Monsoon season showed the highest species richness across all families: Hesperiidae (22 species), Lycaenidae (36 species), Nymphalidae (62 species), Papilionidae (14 species, just one less than pre‐monsoon), Pieridae (20 species), and Riodinidae (5 species). Winter had the lowest species richness across all families. Species abundance was comparatively higher in pre‐monsoon for all families: Hesperiidae (*N* = 52, three fewer than during monsoon), Lycaenidae (*N* = 177), Nymphalidae (*N* = 596), Papilionidae (*N* = 139), Pieridae (*N* = 363), except for Riodinidae, which was highest in post‐monsoon (*N* = 13) (Table 2 and Figure S9). For seasonal species richness and abundance at each site (see Table S8).

**TABLE 2:** Species richness and abundance (*N*) of butterfly families across the seasons.

Family	Pre‐monsoon		Monsoon		Post‐monsoon		Winter	
	Richness	*N*	Richness	*N*	Richness	*N*	Richness	*N*
Hesperiidae	19	52	22	55	9	18	5	11
Lycaenidae	31	177	36	150	21	97	11	58
Nymphalidae	43	596	62	549	40	269	29	233
Papilionidae	15	139	14	82	9	32	3	11
Pieridae	16	363	20	186	13	79	7	144
Riodinidae	4	9	5	21	2	13	0	0

### 3.4 Effects of Monthly Precipitation and Temperature on Butterfly Species Richness and Abundance

**FIGURE 6:**
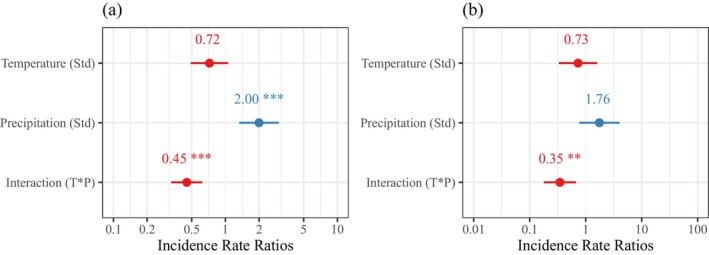
Standardized incidence rate ratios (IRR) for butterfly (a) species richness (Poisson GLMM) and (b) abundance (Negative binomial GLMM). Points represent the estimated IRR with 95% confidence intervals; values > 1.0 indicate a positive effect, while values < 1.0 indicate a negative effect.

We found that monthly precipitation has a significant positive effect on butterfly species richness (*p* < 0.05). Meanwhile, the monthly average temperature had a negative effect, but it was not significant (*p* > 0.05). There was a significant negative interactive effect of temperature and precipitation on species richness (*p* < 0.05). Likewise, precipitation and temperature alone have no significant effect on abundance (*p* > 0.05). In contrast, their interaction has a significant negative effect on abundance (*p* = 0.00168) (Figure 6 and Table 3).

**TABLE 3:** Summary statistics of GLMMs showing the effect of monthly precipitation and average temperature on butterfly species richness and abundance.

Response	Effect	Estimate	Std. error	*z*	*p*
Richness (Poisson)	Intercept	3.8886	0.2886	13.475	< 0.001
	Temperature (Std.)	−0.3279	0.1969	−1.665	0.096
	Precipitation (Std.)	0.691	0.2071	3.336	0.00085
	Temp. × Prec.	−0.7939	0.1637	−4.85	< 0.001
Abundance (Negative binomial)	Intercept	5.0706	0.4233	11.979	< 0.001
	Temperature (Std.)	−0.3135	0.4009	−0.782	0.43426
	Precipitation (Std.)	0.5635	0.4238	1.330	0.18366
	Temp. × Prec.	−1.0582	0.3368	−3.142	0.00168

*Note:* Significant *p*‐values are in bold (*p* < 0.05).

## 4 Discussion

In this study, we explored the diversity and community composition of butterflies across seasons in the urban forests of Pokhara City. The butterflies recorded in our study represent 28.34% of the total butterfly species documented in Nepal (Kc et al. 2025; van der Poel and Smetacek 2022). In the Pokhara Valley, Subedi et al. (2021) documented 138 butterfly species from Rupa Lake, while Kc (2025) reported 225 species in Methlang Forest. Additionally, 174 species had been previously reported from Bengas and Rupa Lakes (Smith et al. 2016). These findings, both previous and recent, signify that the Pokhara Valley supports the rich diversity of butterfly fauna. Using additional survey methods such as bait traps, malaise traps, and square transects (Freitas et al. 2020; Bruschini et al. 2024) might yield a more complete list of butterflies in the study area. Nevertheless, the butterflies we reported serve as a vital baseline for long‐term monitoring and provide crucial information to support management strategies in these urban forests amid urban expansion.

### 4.1 Seasonal Variation of Butterfly Diversity and Community Composition

The diversity and community composition of butterflies varied significantly across seasons, with the monsoon showing the highest and winter the lowest observed species richness, Shannon, and Simpson diversities. This richness and diversity during the monsoon could be attributed to rainfall, which creates favorable conditions for food availability, oviposition, and flight, due to the presence of diverse plant life and young foliage (Júnior and Diniz 2015; Morais et al. 1999). The wet and dry seasons, along with the availability of host plants, play crucial roles in shaping butterfly diversity and seasonal changes (Meléndez‐Jaramillo et al. 2019; Shuey et al. 2024; Valtonen et al. 2013). The pre‐monsoon period showed the highest abundance, likely because larvae emerge as temperature rises and rainfall begins to increase, thriving during the dry season (Janzen 1987). The initial rain triggers heightened insect activity (Wolda 1988). Similarly, seasonal peaks in the abundance of dominant butterfly families and species can lead to higher numbers in certain seasons (Gupta et al. 2019), for example, higher abundance of *P. canidia* and *J. iphita* during pre‐monsoon in our study area.

Our findings on species richness align with previous research in subtropical regions such as Kathmandu and the Syangja Districts (Neupane and Miya 2021; Thapa 2008). Another study in urban forests in Kathmandu also observed the lowest richness and abundance during winter (Shrestha et al. 2018). A two‐season study in Kirtipur reported higher diversity after the monsoon than during it (Oli and Sharma 2019), whereas another study found the opposite (Bhatta 2023). Several other studies in various parts of Nepal's subtropical regions reported different seasonal peaks of richness, diversity, and abundance—for example, richness peaked in pre‐monsoon (Kc 2025; Kc and Sapkota 2024; Nepali et al. 2018), post‐monsoon (Dahal 2017; Rai 2017), and abundance peaked in pre‐monsoon (Rai 2017) and monsoon (Dahal 2017; Miya et al. 2021). The post‐monsoon period—marked by maximum richness, abundance, and diversity—was also noted in a temperate zone (Prajapati et al. 2000). Similarly, in tropical regions, species richness and abundance were higher during pre‐monsoon (Bhusal and Khanal 2009; Miya et al. 2025), monsoon (Tamang et al. 2019), winter (Sah 2019), and post‐monsoon (Oli et al. 2023). This seasonal variation in butterfly diversity across regions may be driven by geographic and bioclimatic factors, as well as vegetation, which affect butterfly species composition (Cómbita et al. 2022).

The monsoon and post‐monsoon periods showed the highest species evenness but had the greatest differences in community composition between them. Species such as *P. canidia*, *E. hecabe*, and *Y. baldus* contributed most to the dissimilarity between these two seasons, as well as across all seasons. A community composition similar to that in our study was also reported in the subtropical lowlands of Bhutan (Singh 2012). Likewise, in the sub‐humid region of Tamaulipas, Mexico, the butterfly community showed high similarity between the early and late rainy seasons (Meléndez‐Jaramillo et al. 2019). The least similarity between pre‐monsoon and post‐monsoon was observed in the tropical forest of Northeast India (Singh et al. 2015). Moreover, the species composition was significantly similar between the rainy and winter seasons in Telangana, Jammu, and Kashmir (Ravivarma et al. 2023; Sharma and Sharma 2021). Additionally, we identified species like *A. emolus* and *L. kansa*, along with several others, as indicators of specific seasons or season combinations, highlighting their dependence on particular seasons.

### 4.2 Seasonal Variation of Family‐Wise Butterfly Species Richness and Abundance

We found that Nymphalidae, followed by Lycaenidae, had the highest species richness, and that Nymphalidae, followed by Pieridae, had the highest abundance in the study area. One reason for this could be that Nymphalidae is one of the most diverse butterfly families, with over 6000 species worldwide (Peña and Espeland 2015; van Nieukerken et al. 2011). Nepal hosts 239 species belonging to this family, which accounts for 34.38% of the country's total recorded species (Kc et al. 2025; van der Poel and Smetacek 2022). Additionally, Nymphalidae are generalists, with a variety of host plants and a wide geographic range (Nylin et al. 2014; Slove and Janz 2011), characterized by active flight, large wingspans, and greater dispersal ability (Freire et al. 2021; Marini‐Filho and Martins 2010). Species such as *J. iphita* and *Y. baldus* from Nymphalidae, along with *P. canidia* and *E. hecabe* from Pieridae, were widespread, contributing to their higher abundance in our study area. Our results align with previous studies from sub‐tropical regions like Rupa Lake (Subedi et al. 2021), Methlang Forest (Kc 2025), urban forests of Kathmandu (Bhatta 2023; Shrestha et al. 2018), and other locations (Kc and Sapkota 2024; Nepali et al. 2018), as well as tropical areas (Khanal 2009; Tamang et al. 2019). Furthermore, similar patterns were observed in temperate zones (Prajapati et al. 2000; Shrestha 2016; Shrestha et al. 2020).

All families showed the greatest species richness during the monsoon and the highest abundance in the pre‐monsoon, with the lowest levels in winter. The monsoon season creates mud‐puddling sites and nectar resources suitable for generalist species, leading to a peak in their diversity (Mahata et al. 2024). Conversely, Riodinidae were most abundant after the monsoon, and Hesperiidae were nearly equally abundant in the pre‐monsoon and monsoon seasons. These findings highlight the seasonal responses of butterfly families in sub‐tropical urban forests. Although the seasonal variation of specific butterfly families has not been studied in the sub‐tropical regions of Nepal, Miya et al. (2025) reported varying responses of butterfly family diversity in tropical lowlands. For example, Hesperiidae showed the highest richness during the monsoon and post‐monsoon seasons, while Lycaenidae, Nymphalidae, and Papilionidae had the highest richness during the pre‐monsoon season. Similarly, Lycaenidae were most abundant in winter, whereas Hesperiidae, Nymphalidae, and Papilionidae peaked in abundance during the pre‐monsoon. The Pieridae remained relatively stable throughout all seasons (Miya et al. 2025).

Likewise, Papilionidae was found to be most abundant in the post‐monsoon season in South India (Arun 2008). In Jharkhand, Pieridae and Papilionidae exhibited slight seasonal variations, while Nymphalidae and Lycaenidae reached their peak richness during the rainy season and were lowest in summer (Verma 2009). Pieridae was most abundant after the monsoon, while Lycaenidae was abundant in winter, and Nymphalidae remained consistently abundant in all seasons except summer in Tamil Nadu (Hussain et al. 2011). Papilionidae and Riodinidae did not show notable seasonal variation, while Hesperiidae were at their highest and Pieridae, Nymphalidae, and Lycaenidae were at their lowest during the rainy season in Kerala's humid climate (Sneha 2018). The rainy season also increased the number of Lycaenidae, Riodinidae, and Hesperiidae in Belize (Shuey et al. 2024). Similarly, Hesperiidae were the richest and most abundant groups in Costa Rica during the dry (humid) season (Murillo‐Hiller et al. 2019). Furthermore, the highest species richness and abundance of Lycaenidae, Nymphalidae, and Pieridae were reported in the rainy season, whereas Papilionidae reached its maximum richness in the dry season in Mexico (Pozo et al. 2008). These findings suggest that different butterfly families show varying seasonality patterns across different climate regions and habitats.

### 4.3 Effects of Monthly Precipitation and Temperature on Species Richness and Abundance

The GLMMs revealed that butterfly richness is positively influenced by precipitation, while temperature alone has no significant effect. However, the interaction between temperature and precipitation significantly negatively impacts both richness and abundance, suggesting that high temperatures may diminish the benefits of precipitation. Precipitation is essential for the availability of nectar and host plants, whereas temperature affects daily activities such as flight and foraging in butterflies (Gullan and Cranston 2014; Kumar et al. 2023). Additionally, temperature indirectly influences butterfly diversity through factors like rainfall, atmospheric pressure, wind, humidity, and vegetation growth (Khanal 2013). Warmer temperature can also create more favorable conditions for generalist butterfly families (Ribeiro and Freitas 2010). In contrast to our findings, a study in tropical lowland Nepal found that precipitation negatively impacts richness and abundance, while temperature positively influences richness (Miya et al. 2025).

Our findings are similar to or differ from several studies worldwide. Both precipitation and temperature were critical climatic factors determining butterfly population dynamics in Europe (Mills et al. 2017). Monthly precipitation and temperature were significantly linked to butterfly richness in Belize (Shuey et al. 2024). Temperature was negatively related to species richness and abundance, while rainfall was positively related to both in coffee–banana agroforests in Uganda (Munyuli 2013). Similarly, in a subtropical habitat in Delhi, India, species richness and abundance were significantly associated with temperature (Gupta et al. 2019). Temperature and relative humidity were the most significant factors for butterfly richness in the tropical dry forest of Eastern Ghats (Mahata et al. 2023). In contrast, no significant link between rainfall and species richness or abundance was found in the Atlantic Forest of Brazil (Ribeiro et al. 2010). In addition to precipitation and temperature, several other factors may affect butterfly populations, including humidity, altitude, habitat types, food plants, and body size (Bhusal and Khanal 2009; Khanal et al. 2013; Pandey et al. 2017).

## 5 Conclusion

The urban forests of Pokhara City host a diverse range of butterfly species belonging to six families. Notable variations in butterfly diversity and community composition were observed across different seasons. Several generalist species contributed to the dissimilarity in community composition between seasons. Species such as *A. emolus* and *L. kansa* were identified to associate with specific seasons. Butterfly richness was highest during the monsoon, while abundance peaked in the pre‐monsoon, and winter showed the lowest levels. The family Nymphalidae, known for its generalist nature, dominated the study area. Butterfly families also exhibited seasonal patterns, with most reaching peak richness in the monsoon and peak abundance in the pre‐monsoon. The interaction between precipitation and temperature significantly influenced both butterfly richness and abundance. Altogether, these findings underscore the importance of seasonality on shaping butterfly diversity, as well as infer that urban forests are crucial habitats for wide range of butterfly species. Future research should consider host plants, other bioclimatic factors, human activities, and include alternative survey methods like bait traps and malaise traps to better predict butterfly diversity in the study area and other urban environments.

## Author Contributions

**Mahamad Sayab Miya:** conceptualization (lead), data curation (lead), formal analysis (lead), funding acquisition (lead), investigation (equal), methodology (lead), project administration (lead), resources (lead), software (lead), supervision (lead), validation (lead), visualization (lead), writing – original draft (lead), writing – review and editing (equal). **Apeksha Chhetri:** conceptualization (supporting), investigation (equal), methodology (supporting), writing – original draft (supporting), writing – review and editing (equal). **Bandana Subedi:** conceptualization (supporting), data curation (supporting), investigation (equal), methodology (supporting), writing – original draft (supporting), writing – review and editing (equal). **Pratiksha Sharma:** data curation (supporting), writing – original draft (supporting), writing – review and editing (equal). **Sanjaya Raj Tamang:** conceptualization (supporting), data curation (supporting), investigation (equal), methodology (supporting), writing – review and editing (equal). **Shristee Panthee:** conceptualization (supporting), data curation (supporting), formal analysis (supporting), investigation (equal), methodology (supporting), supervision (supporting), validation (supporting), writing – original draft (supporting), writing – review and editing (equal). **Hasina Miya:** data curation (supporting), writing – original draft (supporting), writing – review and editing (equal).

## Funding

This study was funded by ENEDAS (Environmental Education and Science), Germany.

## Conflicts of Interest

The authors declare no conflicts of interest.

## Supporting information


**Figure S1:** Upset plot illustrating the number of unique and shared species among three forests. The bottom left horizontal bars represent the total species richness observed at each site. The vertical bars show the size of specific intersections: the number of species found exclusively in the combination of sites marked by the connected black dots below.
**Figure S2:** Rarefaction and extrapolation curves for butterfly species richness and diversity across different forests. (a) Species accumulation curve based on the number of individuals; (b) species richness, (c) exponential Shannon Index, and (d) inverse Simpson Index, all three based on sample coverage. The solid curves represent rarefaction, the dashed lines indicate extrapolation, and the shaded areas show the corresponding 95% confidence intervals. There is no statistically significant difference in the diversity indices between the forests when the confidence intervals overlap at *p* < 0.05.
**Figure S3:** Rarefaction and extrapolation curves for butterfly species richness and diversity across different seasons in Banpale Forest. (a) Species accumulation curve based on the number of individuals; (b) species richness, (c) exponential Shannon Index, and (d) inverse Simpson Index, all three based on sample coverage. The solid curves represent rarefaction, the dashed lines indicate extrapolation, and the shaded areas show the corresponding 95% confidence intervals. There is no statistically significant difference in the diversity indices between seasons when the confidence intervals overlap at *p* < 0.05.
**Figure S4:** Rarefaction and extrapolation curves for butterfly species richness and diversity across different seasons in Bhadrakali Forest. (a) Species accumulation curve based on the number of individuals; (b) species richness, (c) exponential Shannon Index, and (d) inverse Simpson Index, all three based on sample coverage. The solid curves represent rarefaction, the dashed lines indicate extrapolation, and the shaded areas show the corresponding 95% confidence intervals. There is no statistically significant difference in the diversity indices between seasons when the confidence intervals overlap at *p* < 0.05.
**Figure S5:** Rarefaction and extrapolation curves for butterfly species richness and diversity across different seasons in Shanti Ban Batika Forest. (a) Species accumulation curve based on the number of individuals; (b) species richness, (c) exponential Shannon Index, and (d) inverse Simpson Index, all three based on sample coverage. The solid curves represent rarefaction, the dashed lines indicate extrapolation, and the shaded areas show the corresponding 95% confidence intervals. There is no statistically significant difference in the diversity indices between seasons when the confidence intervals overlap at *p* < 0.05.
**Figure S6:** (a) Nonmetric multidimensional scaling (NMDS) ordination of all sampling units (abundance) and (b) Bray–Curtis dissimilarity heatmap, indicating the relative differences in butterfly community composition between seasons in Banpale Forest (*p <* 0.05).
**Figure S7:** (a) Nonmetric multidimensional scaling (NMDS) ordination of all sampling units (abundance) and (b) Bray–Curtis dissimilarity heatmap, indicating the relative differences in butterfly community composition between seasons in Bhadrakali Forest (*p <* 0.05).
**Figure S8:** (a) Nonmetric multidimensional scaling (NMDS) ordination of all sampling units (abundance) and (b) Bray–Curtis dissimilarity heatmap, indicating the relative differences in butterfly community composition between seasons in Shanti Ban Batika Forest (*p <* 0.05).
**Figure S9:** Species richness and abundance of butterfly families across the seasons.
**Table S1:** Hill diversity indices, abundance (*N*), sample coverage (SC), and evenness (*J*) of butterflie*s* across three forests.
**Table S2:** Hill diversity indices, abundance (*N*), sample coverage (SC), and evenness (*J*) of butterflie*s* across seasons in Banpale Forest.
**Table S3:** Hill diversity indices, abundance (*N*), sample coverage (SC), and evenness (*J*) of butterflie*s* across seasons in Bhadrakali Forest.
**Table S4:** Hill diversity indices, abundance (*N*), sample coverage (SC), and evenness (*J*) of butterflie*s* across seasons in Shanti Ban Batika Forest.
**Table S5:** Summary of SIMPER analysis showing the top five butterfly species contributing to community dissimilarity between seasonal pairs.
**Table S6:** Indicator species analysis (IndVal) summary showing butterfly species significantly associated with seasonal combinations.
**Table S7:** Species richness and abundance (*N*) of butterfly families in three forests.
**Table S8:** Species richness and abundance (*N*) of butterfly families across the seasons and forests.

## Data Availability

All data and scripts used for this study are available at the GitHub repository: https://github.com/sayab321/Butterfly.

## Appendix A

List of butterflies documented in the study area (BF = Banpale Forest, BKF = Bhadrakali Forest, SBBF= Shanti Ban Batika Forest) with their scientific name, common name, abundance, and IUCN status (NE = Not Evaluated, LC = Least Concern).

**Table:**

S. No.	Scientific name	Common name	Abundance			IUCN status
			BF	BKF	SBBF	
*a. Hesperiidae*						
1	*Aeromachus jhora* (de Nicéville, 1885)	Gray Scrub Hopper	1	1		NE
2	*Baoris farri* (Moore, 1878)	Paintbrush Swift	1			NE
3	*Caltoris tulsi* (de Niceville, [1884])	Purple Swift	3			NE
4	*Celaenorrhinus leucocera* (Kollar, [1844])	Common Spotted Flat	5			NE
5	*Celaenorrhinus munda* (Moore, 1884)	Himalayan Spotted Flat	3			NE
6	*Gerosis phisara* (Moore, 1884)	Dusky‐Yellow‐breast Flat	1			NE
7	*Hasora badra* (Moore, [1858])	Common Awl	1			NE
8	*Iambrix salsala* (Moore, [1866])	Chestnut Bob	1			NE
9	*Matapa aria* (Moore, [1866])	Common Redeye	9			NE
10	*Matapa purpurascens* (Elwes & Edwards, 1897)	Purple Redeye	2			NE
11	*Matapa sasivarna* (Moore, [1866])	Black‐veined Redeye	1			NE
12	*Notocrypta curvifascia* (C. & R. Felder, 1862)	Restricted Demon	2	1		NE
13	*Odontoptilum angulata* (C. Felder, 1862)	Chestnut Angle	3			NE
14	*Parnara guttatus* (Bremer & Grey, [1852])	Straight Swift	1			NE
15	*Pelopidas sinensis* (Mabille, 1877)	Large‐branded Swift	5			NE
16	*Potanthus pallida* (Evans, 1932)	Pale Dart	4	1		NE
17	*Potanthus pseudomaesa* (Moore, [1881])	Indian Dart	1		2	NE
18	*Pseudoborbo bevani* (Moore, 1878)	Bevan's Swift	4			NE
19	*Pseudocoladenia dan* (Fabricius, 1787)	Fulvous Pied Flat	11	2		NE
20	*Sarangesa dasahara* (Moore, [1866])	Common Small Flat	10	15	12	NE
21	*Spialia galba* (Fabricius, 1793)	Indian Skipper	7	1		NE
22	*Tagiades gana* (Moore, [1866])	Suffused Snow Flat	1			NE
23	*Tagiades litigiosa* (Möschler, 1878)	Water Snow Flat	2	1		NE
24	*Tagiades menaka* (Moore, [1866])	Spotted Snow Flat	3	1	1	NE
25	*Telicota augias* (Linnaeus, 1763)	Pale Palm Dart	6			NE
26	*Telicota bambusae* (Moore, 1878)	Dark Palm Dart	1		2	NE
27	*Udaspes folus* (Cramer, [1775])	Grass Demon	4			NE
28	*Zenonoida eltola* (Hewitson, 1869)	Yellow‐spot Swift	2		1	NE
*b. Lycaenidae*						
29	*Acytolepis puspa* (Horsfield, [1828])	Common Hedge Blue	3		2	NE
30	*Anthene emolus* (Godart, [1824])	Ciliate Blue	2		2	NE
31	*Arhopala amantes* (Hewitson, 1862)	Large Oakblue	8			NE
32	*Arhopala atrax* (Hewitson, 1862)	Indian Oakblue	7			NE
33	*Arhopala centaurus* (Fabricius, 1775)	Centaur Oakblue	3			NE
34	*Arhopala paramuta* (de Nicéville, [1884])	Hooked Oakblue	1	6		NE
35	*Castalius rosimon* (Fabricius, 1775)	Common Pierrot	30	5	5	NE
36	*Catochrysops strabo* (Fabricius, 1793)	Forget‐me‐not	1	1		NE
37	*Celastrina lavendularis* (Moore, 1877)	Plain Hedge Blue	2			NE
38	*Cupido lacturnus* (Godart, [1824])	Indian Cupid	3			NE
39	*Curetis bulis* (Westwood, 1852)	Bright Sunbeam	1			NE
40	*Deudorix epijarbas* (Moore, 1857)	Cornelian	5			NE
41	*Euchrysops cnejus* (Fabricius, 1798)	Gram Blue	3			NE
42	*Heliophorus epicles* (Godart, [1824])	Purple Sapphire	66	2	2	NE
43	*Heliophorus indicus* (Fruhstorfer, 1908)	Indian Purple Sapphire	4			NE
44	*Heliophorus sena* (Kollar, [1844])	Sorrel Sapphire	1			NE
45	*Horaga onyx* (Moore, [1858])	Common Onyx	1			NE
46	*Hypolycaena erylus* (Godart, [1824])	Common Tit	5			NE
47	*Iraota timoleon* (Stoll, [1790])	Silverstreak Blue	1			NE
48	*Jamides alecto* (C. Felder, 1860)	Metallic Cerulean	11		2	NE
49	*Jamides bochus* (Stoll, [1782])	Dark Cerulean	7		1	NE
50	*Jamides celeno* (Cramer, [1775])	Common Cerulean	34	30	23	NE
51	*Lampides boeticus* (Linnaeus, 1767)	Peablue	10	9	2	LC
52	*Lestranicus transpectus* (Moore, 1879)	White‐banded Hedge Blue	7			NE
53	*Loxura atymnus* (Stoll, 1780)	Yamfly	1			NE
54	*Luthrodes pandava* (Horsfield, [1829])	Plains Cupid	6			NE
55	*Prosotas dubiosa* (Semper, [1879])	Tailless Lineblue	10			NE
56	*Prosotas nora* (C. Felder, 1860)	Common Lineblue	10			NE
57	*Pseudozizeeria maha* (Kollar, [1844])	Pale Grass Blue	43	2	9	NE
58	*Rapala manea* (Hewitson, 1863)	Slate Flash	5			NE
59	*Rapala nissa* (Kollar, [1844])	Common Flash	3			NE
60	*Rapala pheretima* (Hewitson, 1863)	Copper Flash	1			NE
61	*Rapala rosacea* de Nicéville, [1889]	Rosy Flash	1			NE
62	*Rapala varuna* (Horsfield, [1829])	Indigo Flash	7			NE
63	*Sinthusa chandrana* (Moore, 1882)	Broad Spark	2			NE
64	*Spalgis epius* (Westwood, 1852)	Apefly	2			NE
65	*Spindasis lohita* (Horsfield, [1829])	Long‐Banded Silverline	3			NE
66	*Spindasis syama* (Horsfield, 1829)	Club Silverline	1			NE
67	*Surendra quercetorum* (Moore, [1858])	Common Acacia Blue	2			NE
68	*Tajuria maculata* (Hewitson, 1865)	Spotted Royal	1			NE
69	*Taraka hamada* (Druce, 1875)	Forest Pierrot	5		1	NE
70	*Tarucus ananda* (de Nicéville, [1884])	Dark Pierrot	3			NE
71	Udara dilecta (Moore, 1879)	Pale Hedge Blue	4			NE
72	*Zeltus amasa* (Hewitson, 1865)	Fluffy Tit	5	2		NE
73	*Zizeeria karsandra* (Moore, 1865)	Dark Grass Blue	31	5		LC
74	*Zizina otis* (Fabricius, 1787)	Lesser Grass Blue	4			LC
75	*Zizula hylax* (Fabricius, 1775)	Tiny Grass Blue	5			LC
*c. Nymphalidae*						
76	*Abrota ganga* (Moore, 1857)	Sergeant Major	2			NE
77	*Aglais caschmirensis* (Kollar, [1844])	Indian Tortoiseshell	10	6	5	NE
78	*Argynnis hyperbius* (Linnaeus, 1763)	Indian Fritillary	4			NE
79	*Ariadne merione* (Cramer, [1777])	Common Castor	3	2		NE
80	*Athyma jina* (Moore, [1858])	Bhutan Sergeant	2			NE
81	*Athyma nefte* (Cramer, [1780])	Color Sergeant	4	2	1	NE
82	*Athyma opalina* (Kollar, [1844])	Himalayan Sergeant	5			NE
83	*Athyma perius* (Linnaeus, 1758)	Common Sergeant	2		1	NE
84	*Athyma ranga* (Moore, [1858])	Blackvein Sergeant	4			NE
85	*Athyma selenophora* (Kollar, [1844])	Staff Sergeant	3			NE
86	*Cethosia biblis* (Drury, [1773])	Red Lacewing	1			NE
87	*Cethosia cyane* (Drury, [1773])	Leopard Lacewing	2			NE
88	*Charaxes aristogiton* (C. & R. Felder, [1867])	Tawny Rajah	1			NE
89	*Charaxes marmax* (Westwood, 1847)	Yellow Rajah	2			NE
90	*Chersonesia risa* (Doubleday, [1848])	Common Maplet	4	2		NE
91	*Cynitia lepidea* (Butler, 1868)	Grey Count	9	17	17	NE
92	*Cyrestis thyodamas* (Boisduval, 1846)	Common Map	3	4	3	NE
93	*Danaus chrysippus* (Linnaeus, 1758)	Plain Tiger	15	2	1	LC
94	*Danaus genutia* (Cramer, [1779])	Common Tiger	17	8	5	NE
95	*Discophora sondaica* (Boisduval, [1836])	Common Duffer	2	1		NE
96	*Doleschallia bisaltide* (Cramer, [1777])	Autumn Leaf	1			NE
97	*Elymnias hypermnestra* (Linnaeus, 1763)	Common Palmfly	6			NE
98	*Elymnias malelas* (Hewitson, 1863)	Spotted Palmfly	1	2		NE
99	*Elymnias vasudeva* (Moore, 1857)	Jezebel Palmfly		1		NE
100	*Euploea core* (Cramer, [1780])	Common Indian Crow	41	10	10	LC
101	*Euploea klugii* (Moore, [1858])	King Crow	6			NE
102	*Euploea mulciber* (Cramer, [1777])	Striped Blue Crow	3		5	NE
103	*Euthalia aconthea* (Cramer, [1777])	Common Baron	1	2		NE
104	*Hestinalis nama* (Doubleday, 1844)	Circe	5	6		NE
105	*Heteropsis malsara* (Moore, 1857)	White‐line Bushbrown	40	6	4	NE
106	*Hypolimnas bolina* (Linnaeus, 1758)	Great Eggfly	9	9	15	NE
107	*Hypolimnas misippus* (Linnaeus, 1764)	Danaid Eggfly	1			LC
108	*Junonia almana* (Linnaeus, 1758)	Peacock Pansy	20	8	5	LC
109	*Junonia atlites* (Linnaeus, 1763)	Grey Pansy	5	3	1	NE
110	*Junonia iphita* (Cramer, [1779])	Chocolate Pansy	129	41	55	NE
111	*Junonia lemonias* (Linnaeus, 1758)	Lemon Pansy	31	35	19	NE
112	*Junonia orithya* (Linnaeus, 1758)	Blue Pansy	2			LC
113	*Kallima inachus* (Boisduval, 1846)	Orange Oakleaf	12		3	NE
114	*Kaniska canace* (Linnaeus, 1763)	Blue Admiral			2	NE
115	*Lethe chandica* (Moore, [1858])	Angled Red Forester	3			NE
116	*Lethe confusa* (Aurivillius, 1898)	Banded Treebrown	7	15	23	NE
117	*Lethe europa* (Fabricius, 1775)	Bamboo Treebrown	2			NE
118	*Lethe isana* (Kollar, [1844])	Common Forester	2			NE
119	*Lethe kansa* (Moore, 1857)	Bamboo Forester	4			NE
120	*Lethe verma* (Kollar, [1844])	Straight‐banded Treebrown			8	NE
121	*Melanitis leda* (Linnaeus, 1758)	Common Evening Brown	14	9	11	LC
122	*Melanitis phedima* (Cramer, [1780])	Dark Evening Brown	1			NE
123	*Mycalesis adamsoni* (Watson, 1897)	Double‐banded Bushbrown	5			NE
124	*Mycalesis francisca* (Stoll, [1780])	Lilacine Bushbrown	20	4	16	NE
125	*Mycalesis mineus* (Linnaeus, 1758)	Dark‐brand Bushbrown	5			NE
126	*Mycalesis perseus* (Fabricius, 1775)	Common Bushbrown	2		3	NE
127	*Mycalesis visala* (Moore, [1858])	Long‐brand Bushbrown	6			NE
128	*Neptis cartica* (Moore, 1872)	Plain Sailer	7			NE
129	*Neptis hylas* (Linnaeus, 1758)	Common Sailer	64	45	30	NE
130	*Neptis magadha* (C. & R. Felder, [1867])	Spotted Sailer	1			NE
131	*Orsotriaena medus* (Fabricius, 1775)	Jungle Brown	68	17	17	NE
132	*Pantoporia hordonia* (Stoll, [1790])	Common Lascar	8	4		NE
133	*Parantica aglea* (Stoll, [1782])	Glassy Tiger	62	11	21	NE
134	*Phaedyma columella* (Cramer, [1780])	Short‐banded Sailer	3			NE
135	*Phalanta phalantha* (Drury, [1773])	Common Leopard	1			LC
136	*Polyura athamas* (Drury, [1773])	Common Nawab	2			NE
137	*Sephisa chandra* (Moore, [1858])	Eastern Courtier	1			NE
138	*Stibochiona nicea* (Gray, 1846)	Popinjay	1			NE
139	*Symbrenthia hypselis* (Godart, [1824])	Spotted Jester	1	1		NE
140	*Symbrenthia lilaea* (Hewitson, 1864)	Common Jester	29	16	12	NE
141	*Tanaecia julii* (Lesson, 1837)	Common Earl	27	20	37	NE
142	*Tirumala limniace* (Cramer, [1775])	Blue Tiger	3	1	4	NE
143	*Tirumala septentrionis* (Butler, 1874)	Dark Blue Tiger	2	2	2	NE
144	*Vagrans egista* (Cramer, [1780])	Vagrant	2			NE
145	*Vanessa cardui* (Linnaeus, 1758)	Painted Lady	1			LC
146	*Vanessa indica* (Herbst, 1794)	Indian Red Admiral	20	22	19	NE
147	*Ypthima baldus* (Fabricius, 1775)	Common Five‐ring	107	29	30	NE
148	*Ypthima huebneri* (Kirby, 1871)	Common Four‐ring	2	3		NE
149	*Ypthima newara* (Moore, [1875])	Newari Three‐ring	3			NE
150	*Ypthima nikaea* (Moore, [1875])	Moore's Five‐ring	2			NE
*d. Papilionidae*						
151	*Graphium agamemnon* (Linnaeus, 1758)	Tailed Jay	12	4	3	NE
152	*Graphium chironides* (Honrath, 1884)	Veined Jay	4			NE
153	*Graphium cloanthus* (Westwood, 1841)	Glassy Bluebottle	2			NE
154	*Graphium doson* (C. & R. Felder, 1864)	Common Jay	2	10	5	NE
155	*Graphium sarpedon* (Linnaeus, 1758)	Common Bluebottle	9	8	11	LC
156	*Papilio arcturus* (Westwood, 1842)	Blue Peacock	1			NE
157	*Papilio bianor* (Cramer, [1777])	Common Peacock	2			NE
158	*Papilio bootes* (Westwood, 1842)	Tailed Redbreast	2			NE
159	*Papilio clytia* (Linnaeus, 1758)	Common Mime	4		1	NE
160	*Papilio demoleus* (Linnaeus, 1758)	Lime Swallowtail	1			NE
161	*Papilio helenus* Linnaeus, 1758	Red Helen	9			LC
162	*Papilio machaon* (Linnaeus, 1758)	Common Yellow Swallowtail	4	3		LC
163	*Papilio memnon* (Linnaeus, 1758)	Great Mormon	11	9	18	NE
164	*Papilio nephelus* (Boisduval, 1836)	Yellow Helen	1	3	2	NE
165	*Papilio paris* (Linnaeus, 1758)	Paris Peacock	1	5	7	NE
166	*Papilio polytes* (Linnaeus, 1758)	Common Mormon	44	25	27	NE
167	*Papilio protenor* (Cramer, [1775])	Spangle	5	4	3	NE
168	*Troides helena* (Linnaeus, 1758)	Common Birdwing	2			LC
*e. Pieridae*						
169	*Appias lyncida* (Cramer, [1777])	Chocolate Albatross	11	1		NE
170	*Catopsilia pomona* (Fabricius, 1775)	Common Emigrant	31	10	6	NE
171	*Catopsilia pomona pomona* (Fabricius, 1775)	Lemon Emigrant	10	1	11	NE
172	*Catopsilia pyranthe* (Linnaeus, 1758)	Mottled Emigrant	18			NE
173	*Cepora nerissa* (Fabricius, 1775)	Common Gull	5	2		NE
174	*Colias fieldii* (Ménétriés, 1855)	Dark Clouded Yellow	2	1		NE
175	*Delias acalis* (Godart, 1819)	Red‐breast Jezebel	7			NE
176	*Delias descombesi* (Boisduval, 1836)	Red‐spot Jezebel	1			NE
177	*Delias eucharis* (Drury, 1773)	Common Jezebel	1			NE
178	*Delias hyparete* (Linnaeus, 1758)	Painted Jezebel	6	3	2	NE
179	*Delias pasithoe* (Linnaeus, 1767)	Red‐base Jezebel	3	4		NE
180	*Eurema blanda* (Boisduval, 1836)	Three‐spot Grass Yellow	7	5	6	NE
181	*Eurema brigitta* (Stoll, [1780])	Small Grass Yellow	3			LC
182	*Eurema hecabe* (Linnaeus, 1758)	Common Grass Yellow	87	29	46	LC
183	*Eurema laeta* (Boisduval, 1836)	Spotless Grass Yellow	12			NE
184	*Gandaca harina* (Horsfield, [1829])	Tree Yellow	2		1	NE
185	*Gonepteryx nepalensis* (Doubleday, 1847)	Himalayan Brimstone	3		1	NE
186	*Hebomoia glaucippe* (Linnaeus, 1758)	Great Orange Tip	2	2		NE
187	*Ixias pyrene* (Linnaeus, 1764)	Yellow Orange Tip	6		1	NE
188	*Leptosia nina* (Fabricius, 1793)	Psyche	1			NE
189	*Pareronia avatar* (Moore, [1858])	Pale Wanderer	2			NE
190	*Pieris brassicae* (Linnaeus, 1758)	Large Cabbage White	22			LC
191	*Pieris canidia* (Linnaeus, 1768)	Indian Cabbage White	224	102	69	NE
192	*Pontia daplidice* (Linnaeus, 1758)	Bath White	3			LC
*f. Riodinidae*						
193	*Abisara fylla* (Westwood, 1851)	Dark Judy	6	6		NE
194	*Abisara neophron* (Hewitson, 1861)	Tailed Judy	11	9	3	NE
195	*Dodona adonira* (Hewitson, 1866)	Striped Punch	4			NE
196	*Dodona dipoea* (Hewitson, 1866)	Lesser Punch	2			NE
197	*Dodona egeon* (Westwood, [1851])	Orange Punch	2			NE

## Appendix B

Images of recorded butterflies from the study area with their identification (in the caption).

Images 1–30. 1—Grey Scrub Hopper | 2—Paintbrush Swift | 3—Purple Swift | 4—Common Spotted Flat | 5—Himalayan Spotted Flat | 6—Dusky‐Yellow‐breast Flat | 7—Common Awl | 8—Chestnut Bob | 9—Common Redeye | 10—Purple Redeye | 11—Black‐veined Redeye | 12—Restricted Demon | 13—Straight Swift | 14—Indian Dart | 15—Bevan's Swift | 16—Fulvous Pied Flat | 17—Common Small Flat | 18—Indian Skipper | 19—Suffused Snow Flat | 20—Water Snow Flat | 21—Spotted Snow Flat | 22—Dark Palm Dart | 23—Grass Demon | 24—Yellow‐spot Swift | 25—Common Hedge Blue | 26—Ciliate Blue | 27—Indian Oakblue | 28—Centaur Oakblue | 29—Hooked Oakblue | 30—Common Pierrot. Photos: M.S. Miya (1, 2, 4, 7, 8, 9, 12, 13, 15, 16, 17, 19, 20, 21, 22, 23, 26, 28, 29, and 30), and B. Subedi (3, 5, 6, 10, 11, 14, 18, 24, 25, and 27).

Images 31–60. 31—Forget‐me‐not | 32—Plain Hedge Blue | 33—Indian Cupid | 34—Bright Sunbeam | 35—Gram Blue | 36—Purple Sapphire | 37—Indian Purple Sapphire | 38—Common Tit | 39—Silverstreak Blue | 40—Metallic Cerulean | 41—Dark Cerulean | 42—Common Cerulean | 43—Peablue | 44—White‐banded Hedge Blue | 45—Yamfly | 46—Plains Cupid | 47—Common Lineblue | 48—Pale Grass Blue | 49—Slate Flash | 50—Common Flash | 51—Copper Flash | 52—Rosy Flash | 53—Indigo Flash | 54—Broad Spark | 55—Apefly | 56—Club Silverline | 57—Common Acacia Blue | 58—Spotted Royal | 59—Forest Pierrot | 60—Pale Hedge Blue. Photos: M.S. Miya (31, 32, 33, 36, 37, 41, 42, 45, 47, 48, 52, 56, 59, and 60), and B. Subedi (34, 35, 38, 39, 40, 43, 44, 46, 49, 50, 51, 53, 54, 55, 57, and 58).

Images 61–90. 61—Fluffy Tit | 62—Dark Grass Blue | 63—Lesser Grass Blue | 64—Sergeant Major | 65—Indian Tortoiseshell | 66—Indian Fritillary 67—Common Castor | 68—Color Sergeant | 69—Himalayan Sergeant | 70—Common Sergeant | 71—Blackvein Sergeant | 72—Staff Sergeant | 73—Red Lacewing | 74—Leopard Lacewing | 75—Tawny Rajah | 76—Common Maplet | 77—Grey Count | 78—Common Map | 79—Plain Tiger | 80—Common Tiger | 81—Common Duffer | 82—Autumn Leaf | 83—Common Palmfly | 84—Spotted Palmfly | 85—Jezebel Palmfly | 86—Common Indian Crow | 87—Striped Blue Crow | 88—Common Baron | 89—Circe | 90—White‐line Bushbrown. Photos: M.S. Miya (65, 66, 67, 70, 71, 73, 75, 76, 77, 78, 79, 80, 81, 83, 84, 85, 87, 88, and 90) and B. Subedi (61, 62, 63, 64, 68, 69, 72, 74, 82, 85, 86, and 89).

Images 91–120. 91—Great Eggfly | 92—Peacock Pansy | 93—Grey Pansy | 94—Chocolate Pansy | 95—Lemon Pansy | 96—Blue Pansy | 97—Orange Oakleaf | 98—Blue Admiral | 99—Angled Red Forester | 100—Banded Treebrown | 101—Bamboo Treebrown | 102—Bamboo Forester | 103—Straight‐banded Treebrown | 104—Common Evening Brown | 105—Dark Evening Brown | 106—Double‐banded Bushbrown | 107—Lilacine Bushbrown | 108—Dark‐brand Bushbrown | 109—Common Bushbrown | 110—Plain Sailer | 111—Common Sailer | 112—Jungle Brown | 113—Common Lascar | 114—Glassy Tiger | 115—Common Leopard | 116—Common Nawab | 117—Popinjay | 118—Spotted Jester | 119—Common Jester | 120—Common Earl. Photos: M.S. Miya (92, 93, 94, 95, 97, 98, 100, 103, 104, 105, 109, 111, 112, 113, 114, 115, 116, 118, 119, and 120) and B. Subedi (91, 96, 99, 101, 102, 106, 107, 108, 110, and 117).

Images 121–150. 121—Blue Tiger | 122—Dark Blue Tiger | 123—Vagrant | 124—Painted Lady | 125—Indian Red Admiral | 126—Common Five‐ring | 127—Common Four‐ring | 128—Newari Three‐ring | 129—Tailed Jay | 130—Glassy Bluebottle | 131—Common Jay | 132—Common Bluebottle | 133—Common Peacock | 134—Tailed Redbreast | 135—Common Mime | 136—Lime Swallowtail | 137—Red Helen | 138—Great Mormon | 139—Paris Peacock | 140—Common Mormon | 141—Spangle | 142—Common Birdwing | 143—Chocolate Albatross | 144—Common Emigrant | 145—Mottled Emigrant | 146—Common Gull | 147—Dark Clouded Yellow | 148—Red‐spot Jezebel | 149—Painted Jezebel | 150—Red‐base Jezebel. Photos: M.S. Miya (122, 125, 126, 127, 135, 136, 139, 140, 142, 147, and 149) and B. Subedi (121, 123, 124, 128, 129, 130, 131, 132, 133, 134, 137, 138, 141, 143, 144, 145, 146, 148, and 150).

Images 151–167. 151—Three‐spot Grass Yellow | 152—Small Grass Yellow | 153—Common Grass Yellow | 154—Spotless Grass Yellow | 155—Tree Yellow | 156—Himalayan Brimstone | 157—Great Orange Tip | 158—Yellow Orange Tip | 159—Pale Wanderer | 160—Large Cabbage White | 161—Indian Cabbage White | 162—Bath White | 163—Dark Judy | 164—Tailed Judy | 165—Striped Punch | 166—Lesser Punch | 167—Orange Punch. Photos: M.S. Miya (152, 153, 155, 158, 161, 163, and 167) and B. Subedi (151, 154, 156, 157, 159, 160, 162, 165, and 166), and A. Chhetri (164).
