# Supplementary material for: Seasonal Variation of Butterfly Diversity in Subtropical Urban Forests of Nepal
Source: Ecol Evol. 2026 Jul 3;16(7):e73936. doi: 10.1002/ece3.73936 (PMC13331753; doi:10.1002/ece3.73936)
Supplement: Supplementary file 1 — Figure S1: Upset plot illustrating the number of unique and shared species among three forests. The bottom left horizontal bars represent the total species richness observed at each site. The vertical bars show the size of specific intersections: the number of species found exclusively in the combination of sites marked by the connected black dots below. Figure S2: Rarefaction and extrapolation curves for butterfly species richness and diversity across different forests. (a) Species accumulation curve based on the number of individuals; (b) species richness, (c) exponential Shannon Index, and (d) inverse Simpson Index, all three based on sample coverage. The solid curves represent rarefaction, the dashed lines indicate extrapolation, and the shaded areas show the corresponding 95% confidence intervals. There is no statistically significant difference in the diversity indices between the forests when the confidence intervals overlap at p < 0.05. Figure S3: Rarefaction and extrapolation curves for butterfly species richness and diversity across different seasons in Banpale Forest. (a) Species accumulation curve based on the number of individuals; (b) species richness, (c) exponential Shannon Index, and (d) inverse Simpson Index, all three based on sample coverage. The solid curves represent rarefaction, the dashed lines indicate extrapolation, and the shaded areas show the corresponding 95% confidence intervals. There is no statistically significant difference in the diversity indices between seasons when the confidence intervals overlap at p < 0.05. Figure S4: Rarefaction and extrapolation curves for butterfly species richness and diversity across different seasons in Bhadrakali Forest. (a) Species accumulation curve based on the number of individuals; (b) species richness, (c) exponential Shannon Index, and (d) inverse Simpson Index, all three based on sample coverage. The solid curves represent rarefaction, the dashed lines indicate extrapolation, and the shaded ar [file ECE3-16-e73936-s001.docx]

**Seasonal Variation of Butterfly Diversity in Subtropical Urban Forests of Nepal**

Mahamad Sayab Miya^1,2,3^*, Apeksha Chhetri^4^, Bandana Subedi^5^, Pratiksha Sharma^6^, Sanjaya Raj Tamang^7^, Shristee Panthee^8,9^, Hasina Miya^10*^


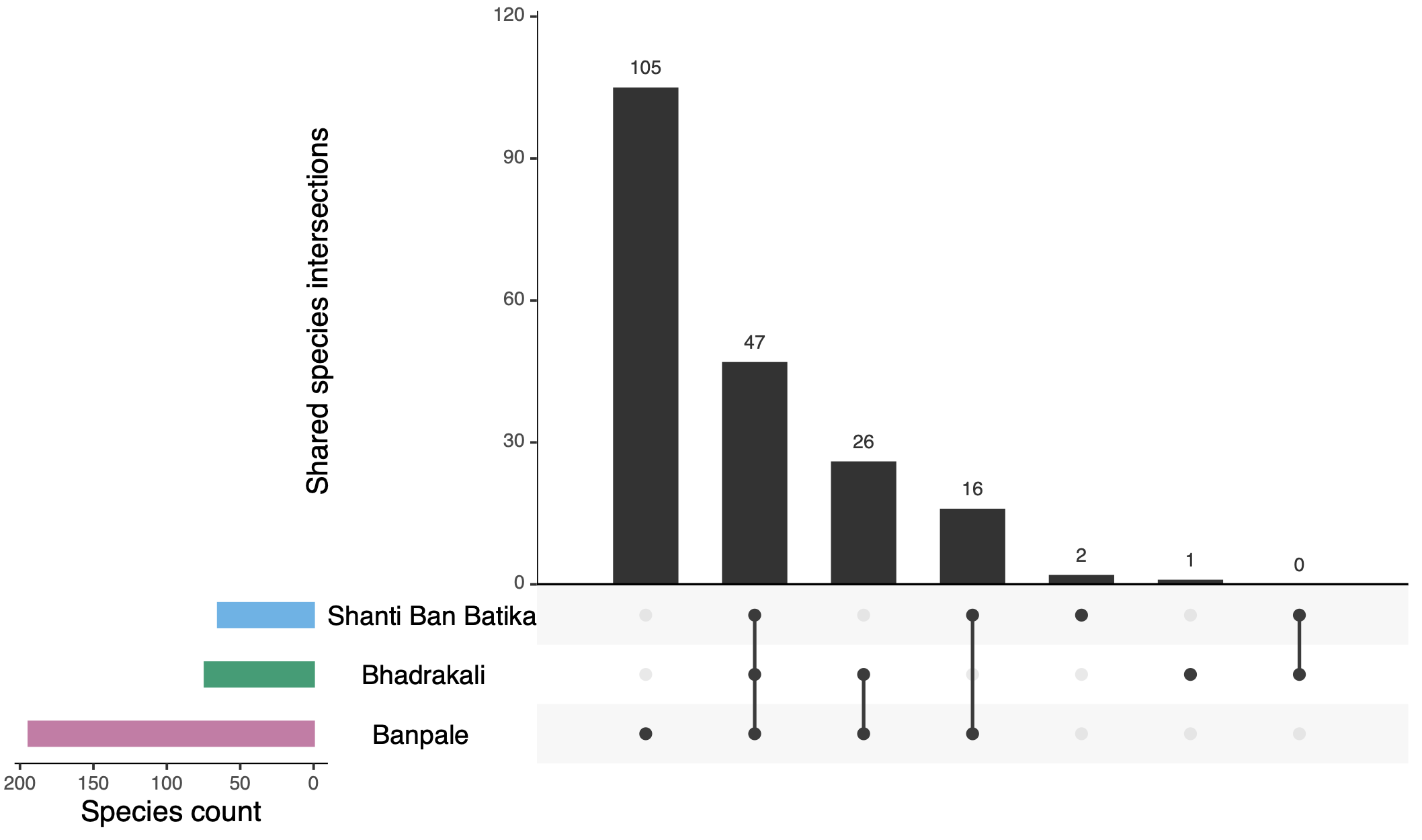


**Figure S1.** Upset plot illustrating the number of unique and shared species among three forests. The bottom left horizontal bars represent the total species richness observed at each site. The vertical bars show the size of specific intersections: the number of species found exclusively in the combination of sites marked by the connected black dots below.


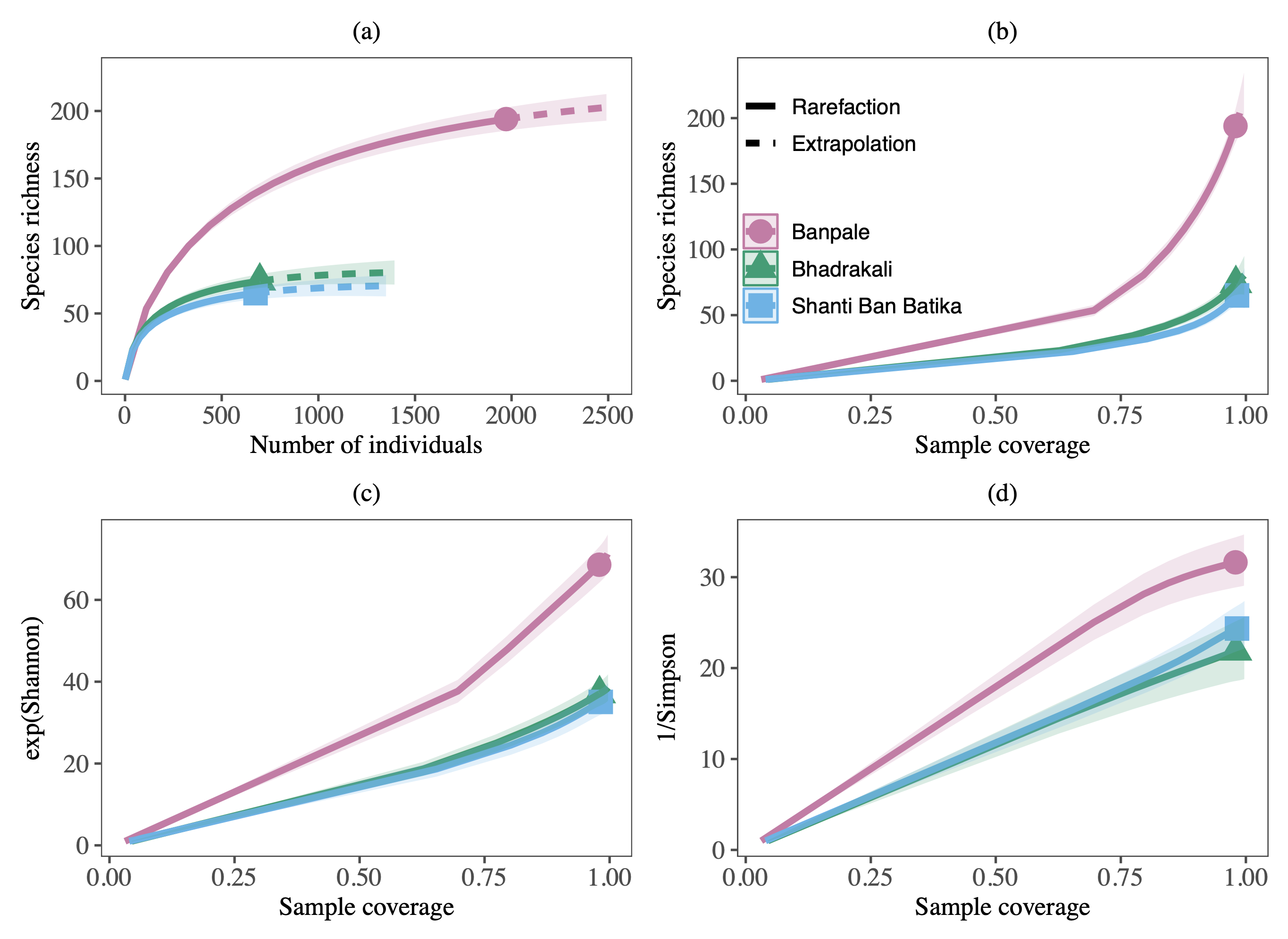


**Figure S2.** Rarefaction and extrapolation curves for butterfly species richness and diversity across different forests. (a) Species accumulation curve based on the number of individuals; (b) species richness, (c) exponential Shannon Index, and (d) inverse Simpson Index, all three based on sample coverage. The solid curves represent rarefaction, the dashed lines indicate extrapolation, and the shaded areas show the corresponding 95% confidence intervals. There is no statistically significant difference in the diversity indices between the forests when the confidence intervals overlap at *p* < 0.05.


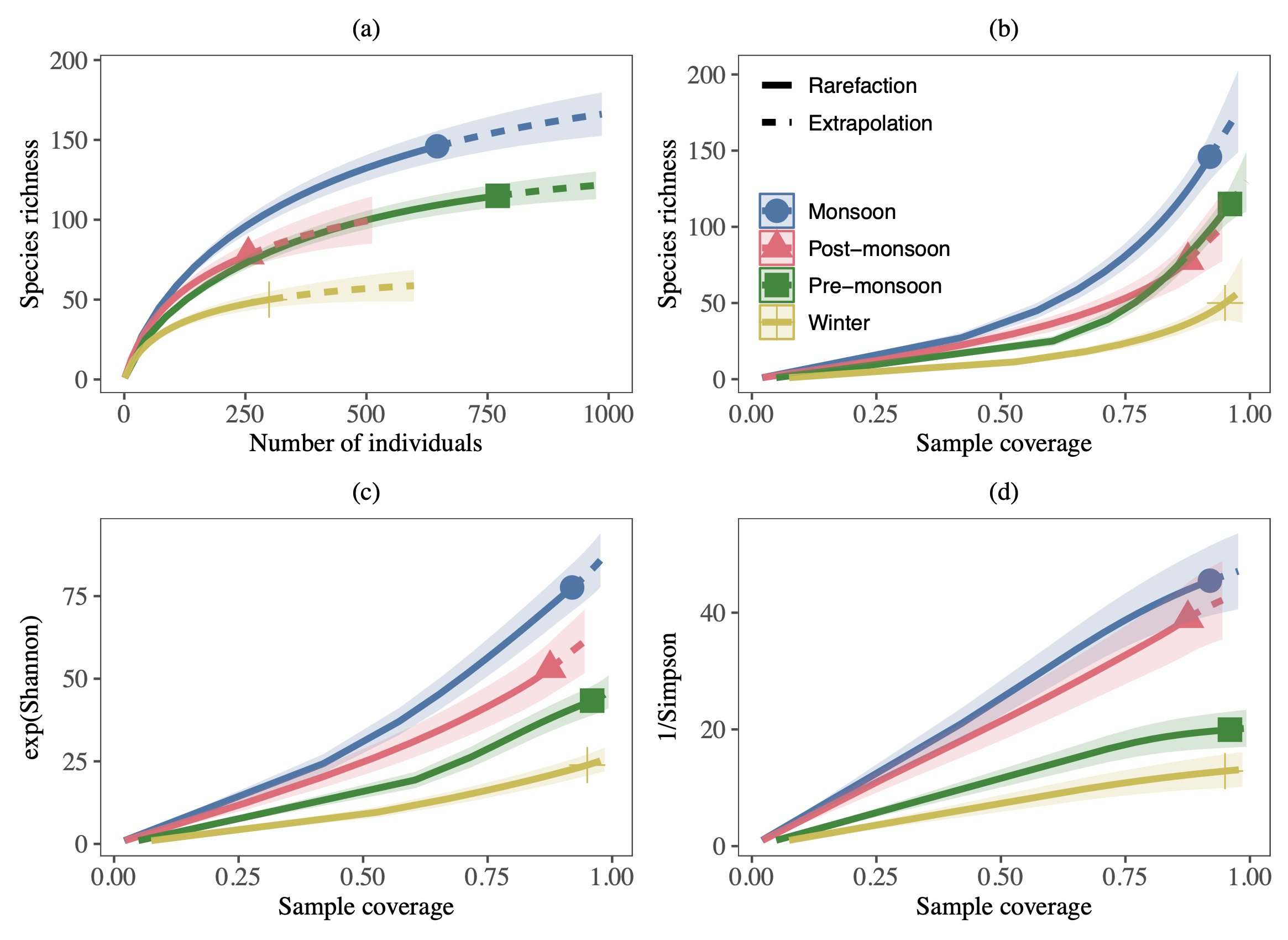


**Figure S3.** Rarefaction and extrapolation curves for butterfly species richness and diversity across different seasons in Banpale Forest. (a) Species accumulation curve based on the number of individuals; (b) species richness, (c) exponential Shannon Index, and (d) inverse Simpson Index, all three based on sample coverage. The solid curves represent rarefaction, the dashed lines indicate extrapolation, and the shaded areas show the corresponding 95% confidence intervals. There is no statistically significant difference in the diversity indices between seasons when the confidence intervals overlap at *p* < 0.05.


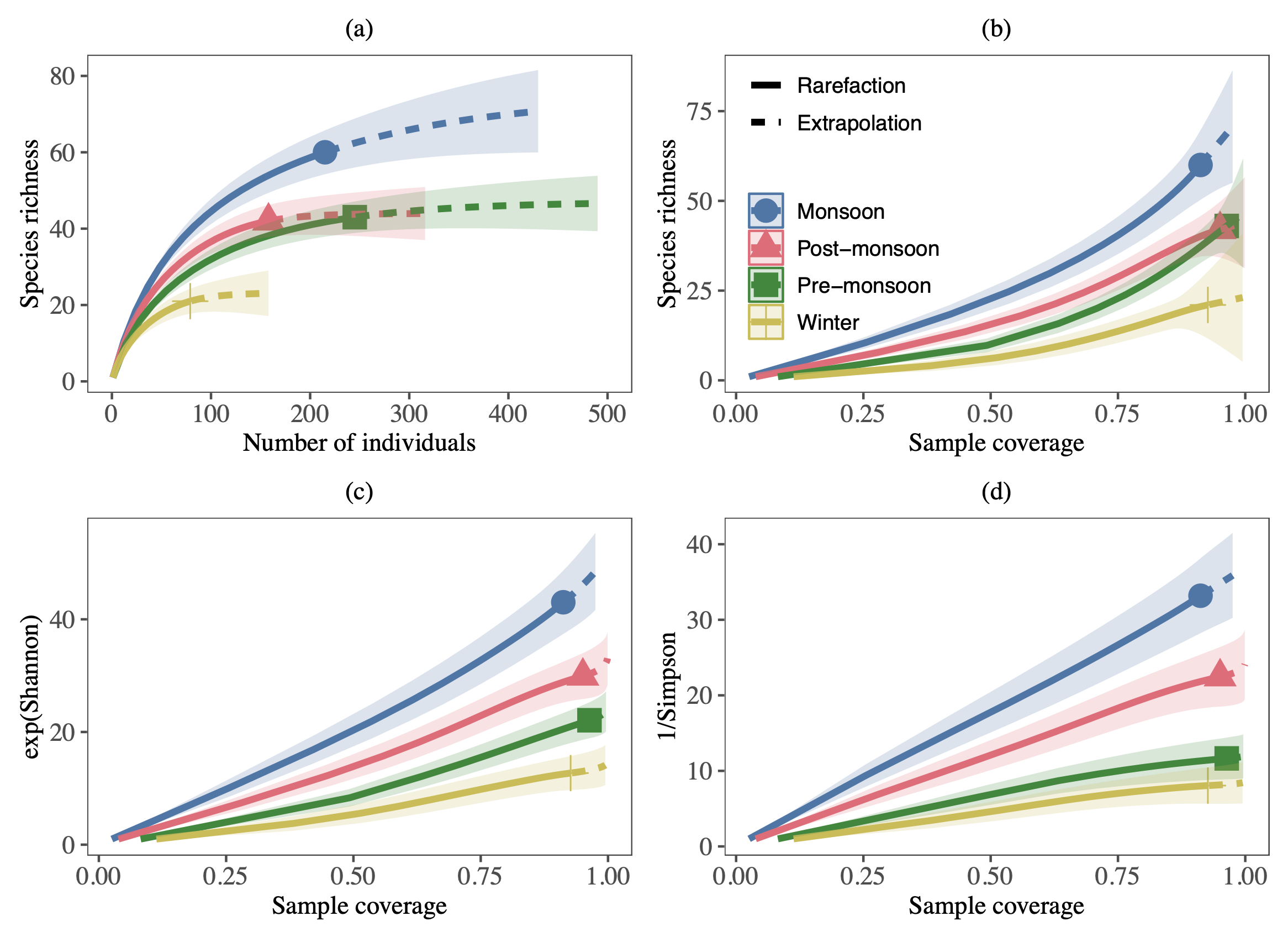


**Figure S4.** Rarefaction and extrapolation curves for butterfly species richness and diversity across different seasons in Bhadrakali Forest. (a) Species accumulation curve based on the number of individuals; (b) species richness, (c) exponential Shannon Index, and (d) inverse Simpson Index, all three based on sample coverage. The solid curves represent rarefaction, the dashed lines indicate extrapolation, and the shaded areas show the corresponding 95% confidence intervals. There is no statistically significant difference in the diversity indices between seasons when the confidence intervals overlap at *p* < 0.05.


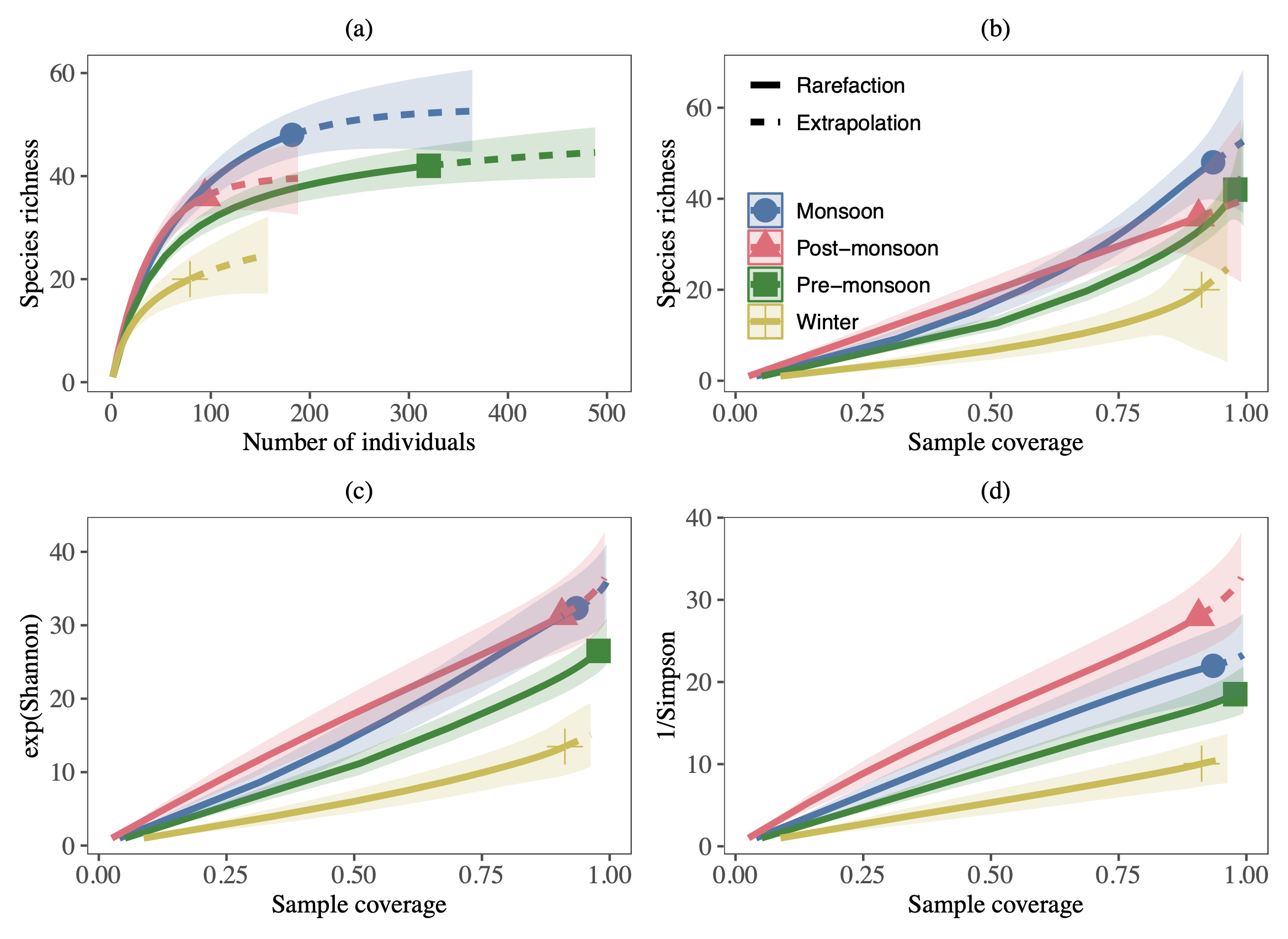


**Figure S5.** Rarefaction and extrapolation curves for butterfly species richness and diversity across different seasons in Shanti Ban Batika Forest. (a) Species accumulation curve based on the number of individuals; (b) species richness, (c) exponential Shannon Index, and (d) inverse Simpson Index, all three based on sample coverage. The solid curves represent rarefaction, the dashed lines indicate extrapolation, and the shaded areas show the corresponding 95% confidence intervals. There is no statistically significant difference in the diversity indices between seasons when the confidence intervals overlap at *p* < 0.05.


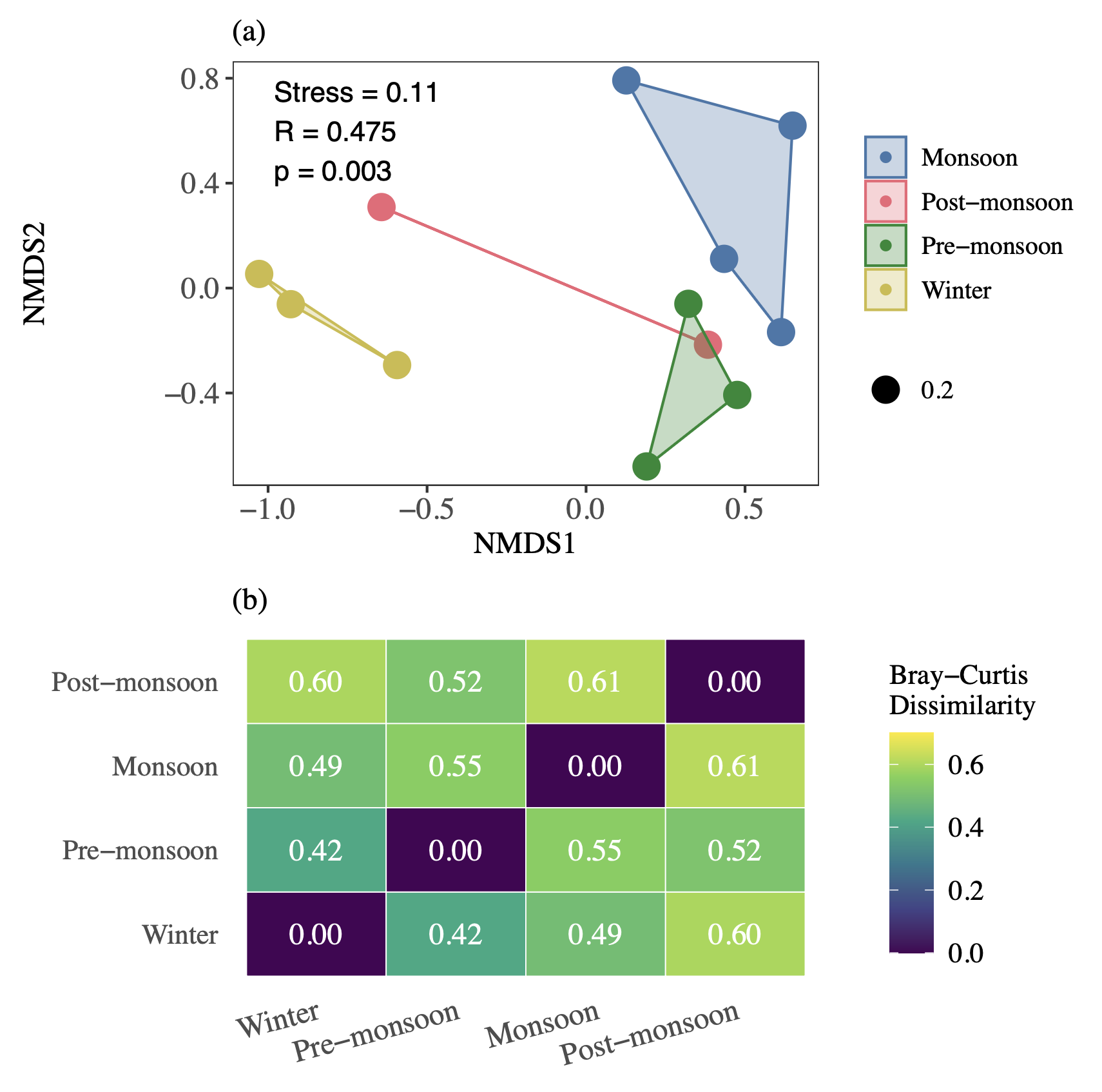


**Figure S6.** (a) Nonmetric multidimensional scaling (NMDS) ordination of all sampling units (abundance) and (b) Bray–Curtis dissimilarity heatmap, indicating the relative differences in butterfly community composition between seasons in Banpale Forest (*p <* 0.05).


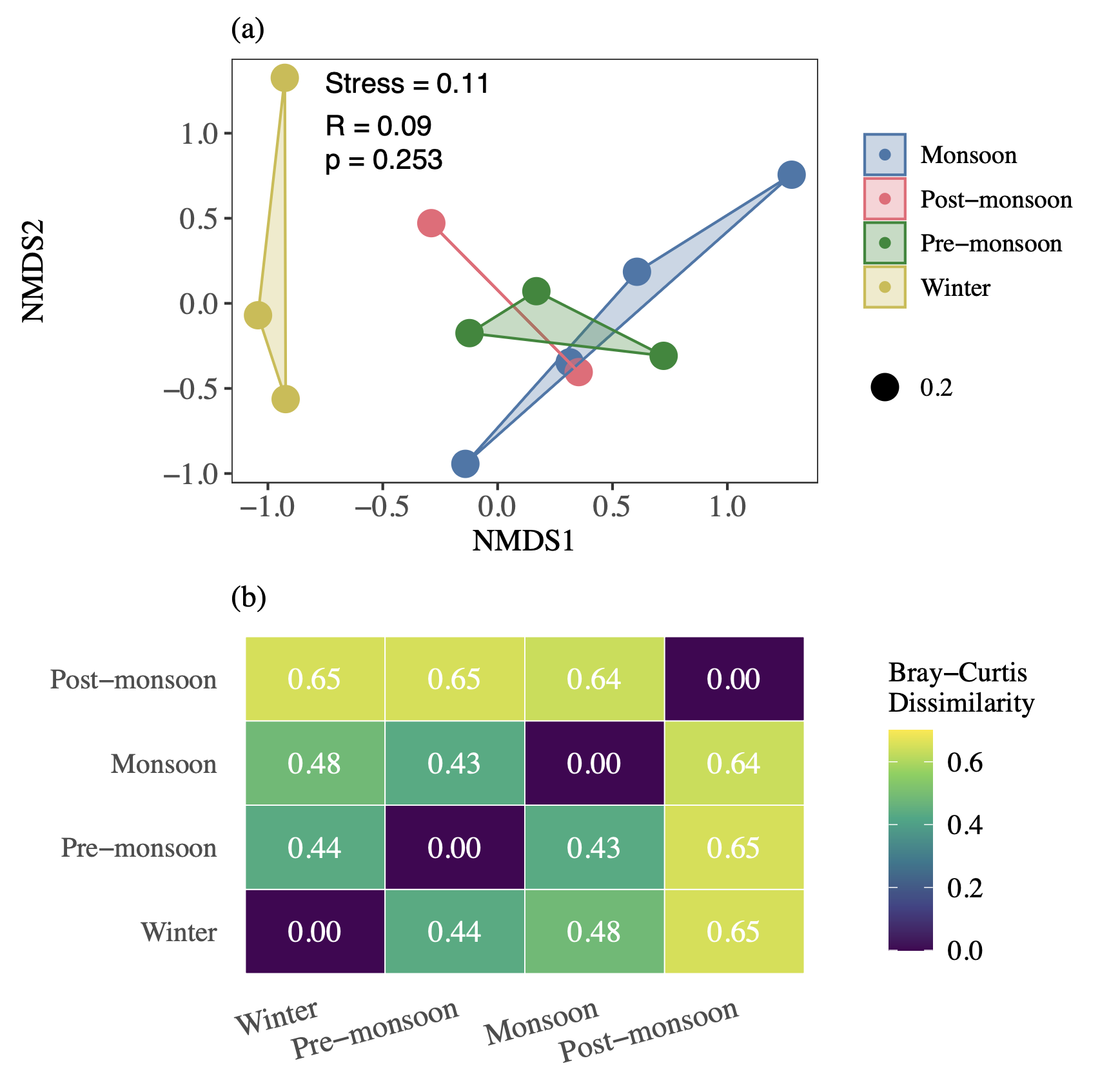


**Figure S7.** (a) Nonmetric multidimensional scaling (NMDS) ordination of all sampling units (abundance) and (b) Bray–Curtis dissimilarity heatmap, indicating the relative differences in butterfly community composition between seasons in Bhadrakali Forest (*p <* 0.05).


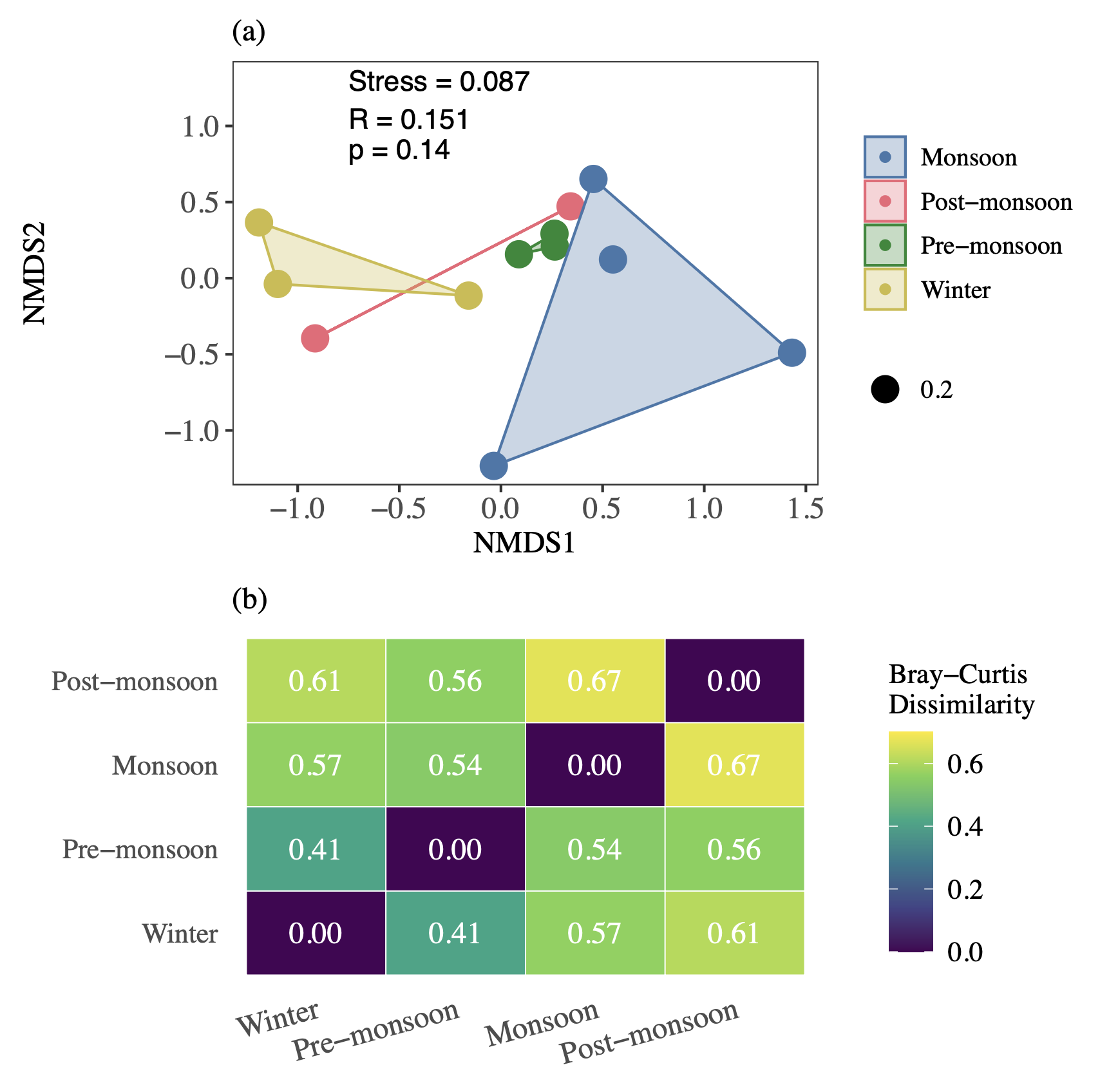


**Figure S8.** (a) Nonmetric multidimensional scaling (NMDS) ordination of all sampling units (abundance) and (b) Bray–Curtis dissimilarity heatmap, indicating the relative differences in butterfly community composition between seasons in Shanti Ban Batika Forest (*p <* 0.05).


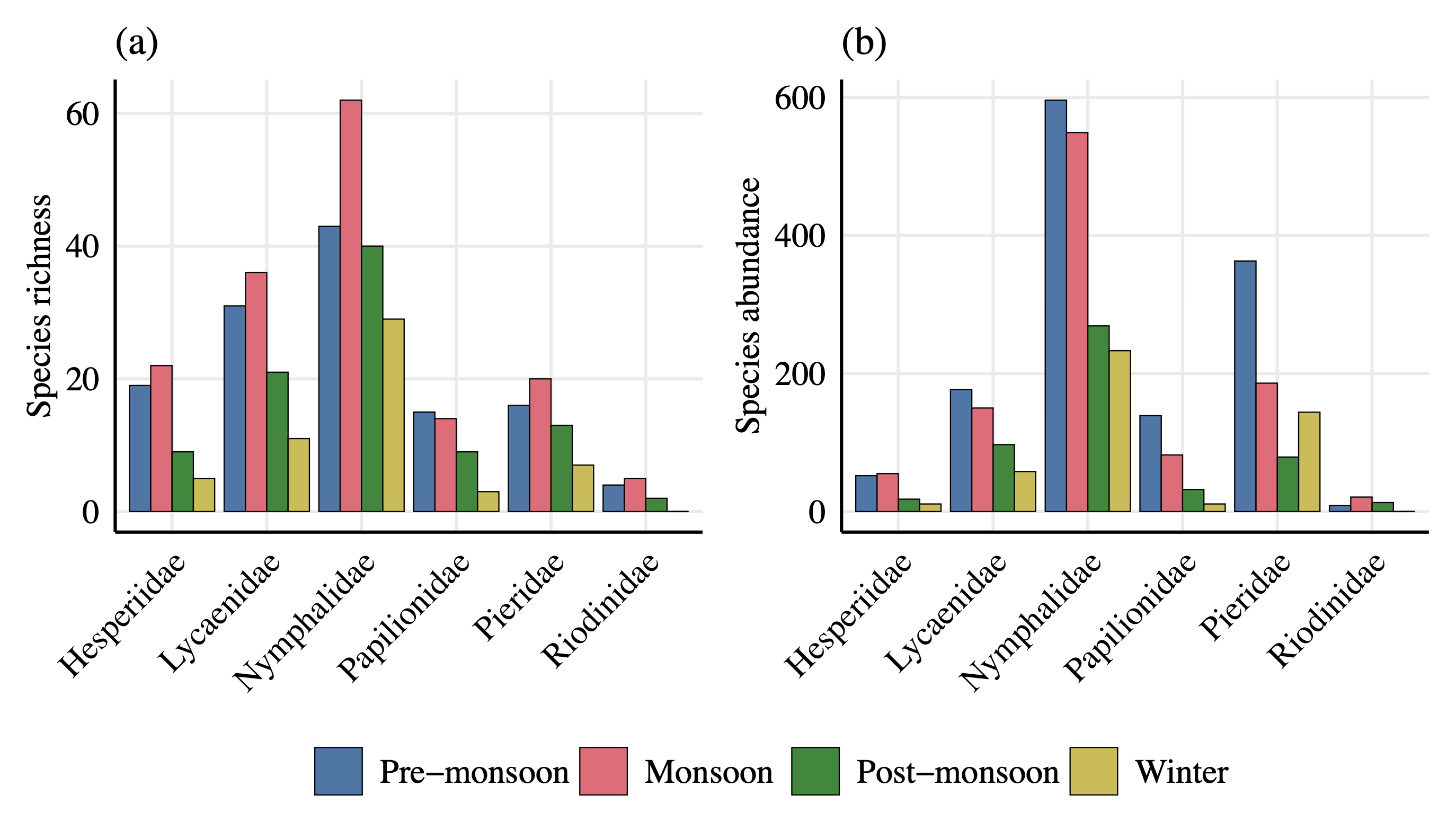


**Figure S9.** Species richness and abundance of butterfly families across the seasons.

**Table S1.** Hill diversity indices, abundance (N), sample coverage (SC), and evenness (J) of butterflies across three forests.

| **Forests** | **Diversity** | **Observed** | **Estimator** | **S.E.** | **LCL** | **UCL** | **N** | **SC** | **J** |
| --- | --- | --- | --- | --- | --- | --- | --- | --- | --- |
| Banpale | Species richness | 194.00 | 218.00 | 12.56 | 194.00 | 242.63 | 1972 | 0.97 | 0.80 |
|  | exp (Shannon) | 68.59 | 72.88 | 2.46 | 68.06 | 77.69 |  |  |  |
|  | 1/Simpson | 31.63 | 32.13 | 1.64 | 28.92 | 35.34 |  |  |  |
| Bhadrakali | Species richness | 74.00 | 81.53 | 9.55 | 74.00 | 100.25 | 697 | 0.98 | 0.84 |
|  | exp (Shannon) | 37.05 | 39.47 | 1.66 | 36.21 | 42.73 |  |  |  |
|  | 1/Simpson | 21.87 | 22.54 | 1.64 | 19.34 | 25.75 |  |  |  |
| Shanti Ban Batika | Species richness | 65.00 | 71.54 | 8.07 | 65.00 | 87.36 | 675 | 0.98 | 0.85 |
|  | exp (Shannon) | 35.06 | 37.11 | 1.44 | 34.29 | 39.94 |  |  |  |
|  | 1/Simpson | 24.35 | 25.22 | 1.41 | 22.45 | 27.99 |  |  |  |

**Table S2.** Hill diversity indices, abundance (N), sample coverage (SC), and evenness (J) of butterflies across seasons in Banpale Forest.

| **Seasons** | **Diversity** | **Observed** | **Estimator** | **S.E.** | **LCL** | **UCL** | **N** | **SC** | **J** |
| --- | --- | --- | --- | --- | --- | --- | --- | --- | --- |
| Pre-monsoon | Species richness | 115.00 | 132.77 | 10.64 | 115.00 | 153.62 | 771 | 0.96 | 0.79 |
|  | exp (Shannon) | 43.38 | 47.74 | 2.89 | 42.08 | 53.39 |  |  |  |
|  | 1/Simpson | 19.96 | 20.46 | 1.86 | 16.82 | 24.10 |  |  |  |
| Monsoon | Species richness | 146.00 | 188.18 | 16.78 | 155.29 | 221.08 | 256 | 0.92 | 0.87 |
|  | exp (Shannon) | 77.61 | 91.40 | 3.78 | 83.99 | 98.81 |  |  |  |
|  | 1/Simpson | 45.48 | 48.85 | 3.47 | 42.05 | 55.65 |  |  |  |
| Post-monsoon | Species richness | 78.00 | 117.23 | 14.17 | 89.46 | 145.00 | 646 | 0.87 | 0.91 |
|  | exp (Shannon) | 53.12 | 68.19 | 4.64 | 59.09 | 77.28 |  |  |  |
|  | 1/Simpson | 39.01 | 45.84 | 4.04 | 37.92 | 53.76 |  |  |  |
| Winter | Species richness | 50.00 | 62.46 | 10.74 | 50.00 | 83.51 | 299 | 0.95 | 0.81 |
|  | exp (Shannon) | 23.87 | 26.77 | 1.85 | 23.14 | 30.39 |  |  |  |
|  | 1/Simpson | 12.86 | 13.39 | 1.49 | 10.48 | 16.31 |  |  |  |

**Table S3.** Hill diversity indices, abundance (N), sample coverage (SC), and evenness (J) of butterflies across seasons in Bhadrakali Forest.

| **Seasons** | **Diversity** | **Observed** | **Estimator** | **S.E.** | **LCL** | **UCL** | **N** | **SC** | **J** |
| --- | --- | --- | --- | --- | --- | --- | --- | --- | --- |
| Pre-monsoon | Species richness | 43.00 | 47.03 | 6.42 | 43.00 | 59.61 | 245 | 0.96 | 0.82 |
|  | exp (Shannon) | 22.09 | 24.51 | 1.85 | 20.87 | 28.14 |  |  |  |
|  | 1/Simpson | 11.65 | 12.18 | 1.52 | 9.20 | 15.16 |  |  |  |
| Monsoon | Species richness | 60.00 | 74.97 | 11.94 | 60.00 | 98.38 | 215 | 0.91 | 0.91 |
|  | exp (Shannon) | 43.02 | 52.21 | 3.25 | 45.83 | 58.58 |  |  |  |
|  | 1/Simpson | 33.18 | 39.06 | 3.48 | 32.23 | 45.88 |  |  |  |
| Post-monsoon | Species richness | 42.00 | 43.99 | 6.93 | 42.00 | 57.57 | 158 | 0.95 | 0.90 |
|  | exp (Shannon) | 29.92 | 34.77 | 2.74 | 29.41 | 40.13 |  |  |  |
|  | 1/Simpson | 22.45 | 26.00 | 2.83 | 20.45 | 31.55 |  |  |  |
| Winter | Species richness | 21.00 | 23.22 | 9.73 | 21.00 | 42.29 | 79 | 0.92 | 0.83 |
|  | exp (Shannon) | 12.71 | 14.91 | 1.90 | 11.18 | 18.63 |  |  |  |
|  | 1/Simpson | 8.05 | 8.85 | 1.76 | 5.40 | 12.30 |  |  |  |

**Table S4.** Hill diversity indices, abundance (N), sample coverage (SC), and evenness (J) of butterflies across seasons in Shanti Ban Batika Forest.

| **Seasons** | **Diversity** | **Observed** | **Estimator** | **S.E.** | **LCL** | **UCL** | **N** | **SC** | **J** |
| --- | --- | --- | --- | --- | --- | --- | --- | --- | --- |
| Pre-monsoon | Species richness | 42.00 | 46.88 | 8.28 | 42.00 | 63.12 | 320 | 0.97 | 0.87 |
|  | exp (Shannon) | 26.51 | 28.65 | 1.51 | 25.69 | 31.62 |  |  |  |
|  | 1/Simpson | 18.52 | 19.59 | 1.63 | 16.39 | 22.79 |  |  |  |
| Monsoon | Species richness | 48.00 | 53.11 | 6.48 | 48.00 | 65.82 | 182 | 0.93 | 0.89 |
|  | exp (Shannon) | 32.32 | 37.95 | 2.64 | 32.78 | 43.11 |  |  |  |
|  | 1/Simpson | 21.97 | 24.84 | 2.77 | 19.41 | 30.28 |  |  |  |
| Post-monsoon | Species richness | 36.00 | 40.01 | 6.55 | 36.00 | 52.84 | 94 | 0.90 | 0.96 |
|  | exp (Shannon) | 31.32 | 39.57 | 3.42 | 32.86 | 46.28 |  |  |  |
|  | 1/Simpson | 27.96 | 39.38 | 4.08 | 31.38 | 47.38 |  |  |  |
| Winter | Species richness | 20.00 | 28.06 | 14.05 | 20.00 | 55.61 | 79 | 0.91 | 0.86 |
|  | exp (Shannon) | 13.48 | 16.28 | 2.46 | 11.47 | 21.09 |  |  |  |
|  | 1/Simpson | 10.05 | 11.37 | 1.92 | 7.59 | 15.14 |  |  |  |

**Table S5.** Summary of SIMPER analysis showing the top five butterfly species contributing to community dissimilarity between seasonal pairs.

| **Species** | **Average dissimilarity** | **SD** | **Ratio** | **A avg.** | **B avg.** | **Cumulative (%)** | ***P*** |
| --- | --- | --- | --- | --- | --- | --- | --- |
| **Monsoon vs post-monsoon** | | | | | | | |
| *P. canidia* | 0.02775 | 0.01743 | 1.592 | 10.25 | 14.5 | 4.86 | 0.988 |
| *J. celeno* | 0.02704 | 0.02398 | 1.12764 | 7.5 | 14 | 9.60 | 0.18 |
| *T. julii* | 0.02380 | 0.02306 | 1.03208 | 14.5 | 4 | 13.78 | 0.262 |
| *E. hecabe* | 0.02265 | 0.01903 | 1.18996 | 15.5 | 8 | 17.75 | 0.361 |
| *Y. baldus* | 0.01699 | 0.01094 | 1.55331 | 11.75 | 14 | 20.73 | 0.836 |
| **Monsoon vs pre-monsoon** | | | | | | | |
| *P. canidia* | 0.09262 | 0.06617 | 1.39956 | 10.25 | 74 | 15.41 | 0.098 |
| *J. iphita* | 0.03924 | 0.02006 | 1.95596 | 15 | 40 | 21.95 | 0.083 |
| *P. polytes* | 0.02060 | 0.01394 | 1.47723 | 4.75 | 18.66666 | 25.38 | 0.078 |
| *Y. baldus* | 0.01817 | 0.01460 | 1.24425 | 11.75 | 20 | 28.40 | 0.81 |
| *H. epicles* | 0.01731 | 0.02266 | 0.76385 | 2.75 | 14.66666 | 31.29 | 0.303 |
| **Monsoon vs winter** | | | | | | | |
| *P. canidia* | 0.07643 | 0.05787 | 1.32072 | 10.25 | 34.33333 | 10.51 | 0.38 |
| *T. julii* | 0.03012 | 0.02901 | 1.03805 | 14.5 | 0.33333 | 14.65 | **0.032** |
| *J. iphita* | 0.02780 | 0.01342 | 2.07111 | 15 | 5 | 18.47 | 0.742 |
| *E. hecabe* | 0.02662 | 0.01580 | 1.68452 | 15.5 | 6 | 22.13 | 0.086 |
| *Y. baldus* | 0.02565 | 0.01776 | 1.44410 | 11.75 | 10.33333 | 25.66 | 0.244 |
| **Post-monsoon vs pre-monsoon** | | | | | | | |
| *P. canidia* | 0.08329 | 0.06351 | 1.31140 | 14.5 | 74 | 14.69 | 0.324 |
| *J. iphita* | 0.03668 | 0.00844 | 4.34178 | 15 | 40 | 21.16 | 0.267 |
| *E. hecabe* | 0.02229 | 0.01732 | 1.28722 | 8 | 22 | 25.09 | 0.372 |
| *J. celeno* | 0.02043 | 0.02024 | 1.0095219 | 14 | 1.33333 | 28.70 | 0.411 |
| *N. hylas* | 0.01772 | 0.00727 | 2.43516 | 9 | 21.33333 | 31.83 | 0.549 |
| **Post-monsoon vs winter** | | | | | | | |
| *P. canidia* | 0.05610 | 0.05231 | 1.07239 | 14.5 | 34.33333 | 9.48 | 0.643 |
| *J. celeno* | 0.03340 | 0.03022 | 1.10520 | 14 | 8.33333 | 15.12 | 0.061 |
| *J. iphita* | 0.02843 | 0.01870 | 1.52031 | 15 | 5 | 19.93 | 0.632 |
| *Y. baldus* | 0.02753 | 0.01385 | 1.98661 | 14 | 10.33333 | 24.58 | 0.216 |
| *E. hecabe* | 0.01979 | 0.00899 | 2.20163 | 8 | 6 | 27.93 | 0.548 |
| **Pre-monsoon vs winter** | | | | | | | |
| *P. canidia* | 0.08195 | 0.06408 | 1.27876 | 74 | 34.33333 | 11.61 | 0.294 |
| *J. iphita* | 0.06089 | 0.01739 | 3.50083 | 40 | 5 | 20.23 | **0.002** |
| *N. hylas* | 0.03131 | 0.01029 | 3.04083 | 21.33333 | 3 | 24.67 | **0.005** |
| *E. hecabe* | 0.02834 | 0.01179 | 2.40389 | 22 | 6 | 28.69 | 0.08 |
| *P. polytes* | 0.02628 | 0.01546 | 1.69997 | 18.66666 | 3 | 32.41 | **0.011** |

*Note*: A and B denote the average abundance of the species in the first and second seasons of each comparison, respectively. Cumulative contributions (%) and significance levels (*p*-values from permutations) refer to the species most responsible for seasonal compositional changes. The significant *p*-values are in bold (*p <* 0.05).

**Table S6.** Indicator species analysis (IndVal) summary showing butterfly species significantly associated with seasonal combinations.

| **Season/Combination** | **Species** | **Indicator value** | ***p*** |
| --- | --- | --- | --- |
| Pre-monsoon | *A. emolus* | 1 | 0.012 |
| Winter | *L. kansa* | 1 | 0.012 |
|  | *A. ranga* | 1 | 0.012 |
| Monsoon + Pre-monsoon | *A. lyncida* | 1 | 0.005 |
|  | *C. pyranthe* | 1 | 0.005 |
| Monsoon + Post-monsoon + Pre-monsoon | *E. core* | 1 | 0.012 |
|  | *P. aglea* | 0.989 | 0.018 |
|  | *Z. karsandra* | 0.979 | 0.015 |
| Post-monsoon + Pre-monsoon | *J. alecto* | 0.973 | 0.006 |
|  | *T. limniace* | 0.95 | 0.009 |
|  | *C. nerissa* | 0.943 | 0.018 |
|  | *H. bolina* | 0.943 | 0.044 |
|  | *T. augias* | 0.933 | 0.021 |
| Monsoon + Post-monsoon | *I. salsala* | 0.913 | 0.021 |
|  | *C. thyodamas* | 0.913 | 0.029 |
|  | *P. helenus* | 0.907 | 0.014 |
|  | *J. bochus* | 0.907 | 0.036 |
|  | *A. opalina* | 0.894 | 0.05 |
|  | *Z. amasa* | 0.877 | 0.025 |
|  | *U. dilecta* | 0.866 | 0.047 |
|  | *E. mulciber* | 0.857 | 0.038 |

*Note*: Only species with statistically significant associations (*p* < 0.05) are shown in the table.

**Table S7.** Species richness and abundance (N) of butterfly families in three forests.

| **Butterfly family** | **Banpale** | | **Bhadrakali** | | **Shanti Ban Batika** | | **Total** | |
| --- | --- | --- | --- | --- | --- | --- | --- | --- |
|  | **Richness** | **N** | **Richness** | **N** | **Richness** | **N** | **Richness** | **N** |
| Hesperiidae | 28 | 95 | 8 | 23 | 5 | 18 | 28 | 136 |
| Lycaenidae | 47 | 371 | 9 | 62 | 10 | 49 | 47 | 482 |
| Nymphalidae | 72 | 896 | 35 | 366 | 31 | 385 | 75 | 1647 |
| Papilionidae | 18 | 116 | 9 | 71 | 9 | 77 | 18 | 264 |
| Pieridae | 24 | 469 | 11 | 160 | 9 | 143 | 24 | 772 |
| Riodinidae | 5 | 25 | 2 | 15 | 1 | 3 | 5 | 43 |

**Table S8.** Species richness and abundance (N) of butterfly families across the seasons and forests.

|  |  | **Pre-monsoon** | | **Monsoon** | | **Post-monsoon** | | **Winter** | |
| --- | --- | --- | --- | --- | --- | --- | --- | --- | --- |
| **Forest** | **Family** | **Richness** | **N** | **Richness** | **N** | **Richness** | **N** | **Richness** | **N** |
| Banpale | Hesperiidae | 16 | 33 | 19 | 41 | 9 | 16 | 4 | 5 |
|  | Lycaenidae | 30 | 155 | 36 | 110 | 20 | 68 | 10 | 38 |
|  | Nymphalidae | 39 | 316 | 55 | 311 | 31 | 113 | 27 | 156 |
|  | Papilionidae | 12 | 60 | 12 | 40 | 5 | 12 | 2 | 4 |
|  | Pieridae | 15 | 203 | 19 | 131 | 11 | 39 | 7 | 96 |
|  | Riodinidae | 3 | 4 | 5 | 13 | 2 | 8 | 0 | 0 |
| Bhadrakali | Hesperiidae | 2 | 8 | 5 | 9 | 1 | 2 | 3 | 4 |
|  | Lycaenidae | 3 | 9 | 6 | 27 | 4 | 19 | 3 | 7 |
|  | Nymphalidae | 23 | 114 | 31 | 124 | 20 | 87 | 11 | 41 |
|  | Papilionidae | 8 | 32 | 8 | 20 | 8 | 18 | 1 | 1 |
|  | Pieridae | 5 | 77 | 8 | 30 | 7 | 27 | 3 | 26 |
|  | Riodinidae | 2 | 5 | 2 | 5 | 2 | 5 | 0 | 0 |
| Shanti Ban Batika | Hesperiidae | 3 | 11 | 3 | 5 | 0 | 0 | 2 | 2 |
|  | Lycaenidae | 5 | 13 | 6 | 13 | 5 | 10 | 2 | 13 |
|  | Nymphalidae | 21 | 166 | 25 | 114 | 24 | 69 | 13 | 36 |
|  | Papilionidae | 7 | 47 | 8 | 22 | 2 | 2 | 1 | 6 |
|  | Pieridae | 6 | 83 | 5 | 25 | 5 | 13 | 2 | 22 |
|  | Riodinidae | 0 | 0 | 1 | 3 | 0 | 0 | 0 | 0 |
